# Data-Driven Robotic Manipulation of Cloth-like Deformable Objects: The Present, Challenges and Future Prospects

**DOI:** 10.3390/s23052389

**Published:** 2023-02-21

**Authors:** Halid Abdulrahim Kadi, Kasim Terzić

**Affiliations:** School of Computer Science, University of St Andrews, Jack Cole Building, North Haugh, St Andrews KY16 9SX, UK

**Keywords:** robotics, cloth-like deformable objects, deep reinforcement learning, deep imitation learning, human–robot interaction, knot theory, general embodied AI

## Abstract

Manipulating cloth-like deformable objects (CDOs) is a long-standing problem in the robotics community. CDOs are flexible (non-rigid) objects that do not show a detectable level of compression strength while two points on the article are pushed towards each other and include objects such as ropes (1D), fabrics (2D) and bags (3D). In general, CDOs’ many degrees of freedom (DoF) introduce severe self-occlusion and complex state–action dynamics as significant obstacles to perception and manipulation systems. These challenges exacerbate existing issues of modern robotic control methods such as imitation learning (IL) and reinforcement learning (RL). This review focuses on the application details of data-driven control methods on four major task families in this domain: cloth shaping, knot tying/untying, dressing and bag manipulation. Furthermore, we identify specific inductive biases in these four domains that present challenges for more general IL and RL algorithms.

## 1. Introduction

Manipulation of cloth-like deformable objects is a critical challenge in robotics and it could be transformative for helping robots play a more active role in people’s daily lives. Robots could free humans from performing house chores such as making beds, folding laundry and tidying up the wardrobe. An agent with bag manipulation skills could help humans with grocery shopping and a dressing-capable agent could provide medical care to elderly or disabled people by helping them dress [1]. Rope-manipulation systems could also perform high-precision operations in medical surgeries [2] and handle cables in factories.

In a typical robotics scenario, an agent controls its actuators to change the environment toward a goal configuration. Even though low-level controllers and motion planning algorithms are robust enough to drive the joints and end-effectors to reach their desired configuration, robots still struggle to solve high-level tasks, such as locomotion [3], autonomous driving [4] and dexterous manipulation [5]. In recent years, data-driven approaches with deep learning (DL) [6], such as deep reinforcement learning (DRL) [7] and deep imitation learning (DIL) [8], have demonstrated many advantages in performing task-level and skill-level control problems (Section 3.3). Nevertheless, manipulating deformable objects using such data-driven approaches remains a challenge due to the large number of degrees of freedom (DoF) associated with the task.

Classical control methods have been successfully applied to CDO manipulation [9,10], but they tend to be restricted to a narrow range of defined configurations. In contrast, data-driven approaches allow to develop more robust and general controllers that can be applied in open environments with a wider range of contextual configurations (Section 3.2). IL and RL are the two candidate approaches for developing such robust controllers that have been explored for cloth shaping (Section 6.1.2) and dressing tasks (Section 6.3.2).

Several surveys and reviews have covered modelling deformable objects and robotic applications. Arriola-Rios et al. (2020) [11] introduce modelling methods of deformable objects (DOs). Sanchez et al. (2018) [12] introduce the categorisation and modelling methods of DOs in general, and they also discuss the difference among the conventional perception and manipulation methods (before 2018) in various DO manipulation tasks [13,14,15,16]. Zhu et al. (2022) [17] discuss the challenges posed by different types of DO manipulation in terms of perception, modelling and manipulation. Borràs et al. (2020) [18] summarise grasping types and end-effectors used in 2D CDO literature and propose a notation system for different grasping types for target objects. Covering modelling, perception and traditional and data-driven control approaches, Yin et al. (2021) [19] provide a similar review but broaden their lens to include the DO domain as a whole.

The difference between our review and all existing reviews is that we focus on cloth-like deformable object manipulation tasks from the DRL and DIL points of view. We introduce the general state-of-the-art (SOTA) development of these two control methods in robotics in addition to detailing their applications in the CDO domain. This review also identifies specific challenges for DRL and DIL algorithms, which are especially pertinent in CDO manipulation due to the complex behaviour of CDOs. We hope this review can provide a comprehensive snapshot of the current state of the art in this field and function as a development manual for the future progression of the field. In addition to the four major task families in this review, there are also some other tasks in the literature, including cloth hanging [20], bed making [21], ironing [22], rope insertion [23] and suturing [24]. Due to the limited number of relevant publications, we do not cover these in our review.

**Figure 1:**
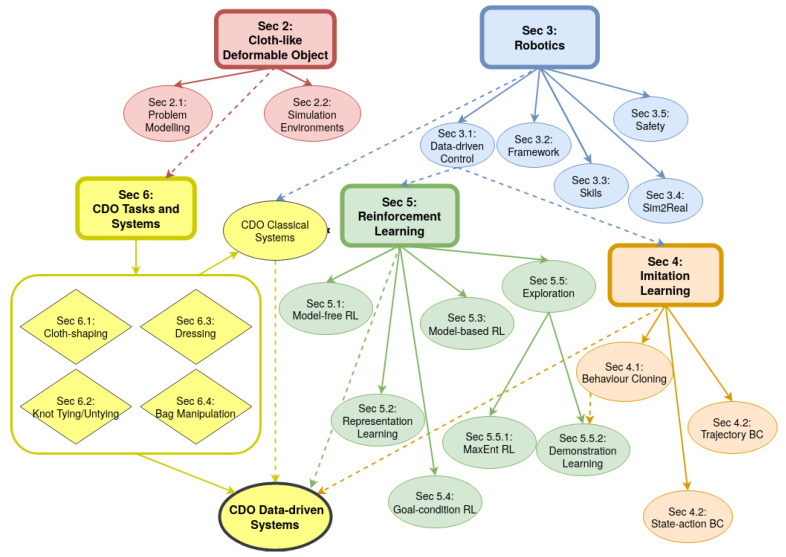
Relationships between different chapters in this review: colours represent individual chapters; solid arrows indicate subsections while dashed arrows show the connection between related subjects.

Figure 1 illustrates the structure of the review. In Section 2, we define CDOs and modelling methods used in modern simulators. Section 3 covers common robotics approaches while highlighting the relation to CDO systems. It covers the development from classical robotics to deep robotics (Section 3.1) and presents a general framework of single-agent robotics that provides mathematical foundations for IL and RL (Section 3.2). Section 4 and Section 5 summarise the theory, challenges and SOTA development of IL and RL algorithms that are commonly applied in the CDO domain. In Section 6, we introduce existing classical and data-driven robotic manipulation systems (RMSs) in four prevailing task families in CDO manipulation literature, including cloth shaping, knot tying/untying, dressing and bag manipulation; we also identify specific challenges in each of these task families. Finally, Section 7 summarises and discusses the inductive biases in these four domains and indicates the future research direction of data-driven approaches for CDO manipulation.

## 2. Models of Cloth-like Deformable Objects

Sanchez et al. (2018) [12] define a deformable object (DO) as an object that undergoes deformation (change of shape) when subjected to external forces [14,15]. They classify DOs into linear, planar and solid DOs based on the number of dimensions used to describe them. They name planar DOs that do not exhibit compression strength under opposing external forces as cloth-like deformable objects (CDOs). In this review, we also consider linear deformable objects that have such properties as a special case of CDOs. Thus, in our classification, CDOs can be one-dimensional (ropes, wires, cables and threads), two-dimensional (towels, fabrics and bedsheets) and three-dimensional (T-shirts, trousers, bags and sacks).

The first challenge in DO manipulation and simulation is to select an appropriate representation for the target object, where the major criteria are expressiveness, accuracy and flexibility [11] of the representation. The next challenge is to model the dynamic behaviour of DOs, where computational complexity, physical accuracy and implementation simplicity are the important criteria for assessing the practicality of a dynamic model. Approaches for modelling dynamics of DOs include mass-spring, particle-based, position-based, constitutive, and neural network systems [11]. We refer to Arriola-Rios et al. (2020) [11] and Yin et al. (2021) [19] for details on these representation and modelling methods.

### 2.1. Problem Modelling

Apart from the compression strength mentioned above, many other physical properties describe the deformation of an object. Plasticity refers to permanent deformation caused by an external force $f_{e}$. Elastic deformation, on the other hand, means that the object can return to its original shape after removing the applied force. Elastoplasticity is a combination of both elastic and plastic deformations, where the object partially returns to its original shape [12]. Stiffness refers to how much force one needs to apply to cause deformation on an object. Furthermore, the degree of deformation caused by stress is called strain ϵ. Hooke’s law relates stress and strain with Young’s modulus *E* that is determined by the material properties of the object σ=Eϵ, where *E* is a constant that can also be expressed with Poisson’s ratio ν; and stress σ is the ratio between the external force and the cross-section area [25]. The formula is often used in simulations to calculate deformations at the element level of DOs, such as in position-based and constitutive systems.

### 2.2. Simulation Environments

Many simulation environments are used to model CDOs in literature, and are seen as essential for the development of DRL and DIL controllers. *Nvidia Flex* [26,27] is an open-source physics engine that uses *Position-Based Dynamics (PBD)* [28] to unify the dynamics of different types of objects; Lin et al. (2021) [29] developed *SoftGym* for cloth and liquid manipulation tasks using the engine. *Nvidia PhysX* [30] is another physics engine that supports DO simulation using *Finite Element Method (FEM)*; robotic literature in the dressing task family has leveraged *PhysX* for training RL agents [31,32].

*Pybullet* [33] is a robotic simulator that builds upon *Bullet* physics engine that can simulate rigid and deformable objects; Seita et al. (2021) [34] developed a cloth shaping and bag manipulation benchmark environment *DeformableRaven* based on this simulator. *Blender* is an open-source 3D creation suite built on *Bullet* and it uses the *Mass–Spring–Damper (MSD)* system to simulate the prescribed dynamics of CDO [35]; however, it is primarily used for generating synthetic data [36] and rendering of CDOs [21,37,38] as it cannot support online interaction.

Other simulators are being leveraged to develop perception and/or control systems for CDO manipulation. *Multi-Joint dynamics with Contact (Mujoco)* [39] provides a robotics toolkit, which supports CDO simulation via soft constraints. *Simulation Open Framework Architecture (SOFA)* [40] physics engine is developed for medical simulation, which uses FEM to model DO and can model the interaction between rigid and deformable objects and allows customised solvers for different mechanical objects, constraints and collision geometries. *Unity with Obi* [41] has been used by Weng et al. (2021) [42] for generating synthetic data for bag manipulation and it could provide a good commercial environment for rope manipulation [43]. *ARCsim* [44] is a cloth-specific simulation environment which has been employed in many perception-focused applications [45,46].

None of the simulators can model the interaction between the cloth and the gripper—instead, *Nvidia Flex* and *Pybullet* “anchor” the cloth to the gripper (marking certain particles as gripped so that rest of the cloth moves accordingly). *DeformableRaven* models a rope and the hem of a sack using a chain of beads, which introduces a significant reality gap. Other than improving the fidelity of the current benchmark environments, the community also needs standard environments for knot-tying/untying and dressing tasks. Improving the simulation and creating more skill-related benchmark environments could accelerate progress in this field.

## 3. Fundamentals of Robotics for CDO Manipulation

Following Kroemer et al. (2021) [47], this section focuses on providing the formalisation of a single centralised decision-making rigid-body agent. For specific details on existing CDO manipulation systems, see Section 6. The minimum requirement for defining a robot is having actuators that it can control [48]. The objective of a robotic manipulation system (RMS) is to interact with its environment to change it to a specified goal configuration g.

Earliest RMSs perform tasks with prescribed motion phases and analytical models to generate low-level control signals. Such systems are only practical in a closed and deterministic environment. In order to perform tasks in more stochastic environments, perception systems play a critical role in updating the robot’s understanding of the configuration and changes in the environment [49,50]. Classical robotics often defines a clear separation between perception and control where the two procedure communicate through explicit intermediate representations. Perception is often handcrafted, as is the control procedure. Control systems in robotics are often hierarchical, with the highest-level action abstraction often prescribed by a heuristic, and lower-level control actions are delivered using motion planning and low-level controllers, such as PID and compliant controllers. The conversion between high-level action abstraction and low-level control signals is also often handcrafted in these systems. Details about classical robotics in CDO manipulation for individual domains will be discussed in Section 6.1.1, Section 6.2.1, Section 6.3.1 and Section 6.4.1.

A dynamic model is sometimes involved to provide a prior estimate of the true state of the environment. It can also provide faster future roll-outs for planning/trajectory optimisation methods. The dynamical model often comes in the form of an approximated analytical model [51,52]. This appears in the early examples of RL methods, also known as planning/trajectory optimisation (Section 5.3) in classical robotics [53,54].

Robotic applications often use a hierarchical structure for the action output: a long-horizon multi-step task can be decomposed into a sequence of subtasks. Some fundamental skills (Section 3.3) are used repeatedly across the subtasks. These skills can be further divided into multiple goal-conditioned phases that can be delivered by motion planning algorithms and low-level analytical controllers [47]. Such hierarchical action structures often require a corresponding hierarchical structure at the state representation [47].

### 3.1. Data-Driven Control

Building an analytical model for producing control signals is a difficult process, especially for CDOs due to their complex deformation behaviours. Controllers developed under classical robotics typically only apply to a fixed or a narrow range of configurations [49,50]. One of the key features of modern robotics methods is that they formulate the manipulation problem as a *Partially Observable Markov Decision Process* (Section 3.2) and leverage *Contextual Markov Decision Processes* (Section 3.2) for developing more robust and general skill controllers (Section 3.3). Section 6.1.2, Section 6.2.2, Section 6.3.2 and Section 6.4.1 will detail current applications of data-driven approaches in the CDO domain.

With the advance of deep learning (DL) [6] and high-performance hardware, almost all parts of an RMS can be practically replaced with neural networks (NNs) and trained on collected data. NNs are also convenient and effective at combining multi-modal sensory inputs [55], as well as integrating passive and interactive perception [56,57,58]. We refer to this evolution as the beginning of modern robotics because NNs fundamentally improved the capability of all parts of the system and revolutionised the system design process [7]; however, there is still a clear separation between perception and control—the selection of state representation is still an important step in the system design. In this era, IL using real-world demonstration data has become one of the most popular and robust control algorithms in research and industry [59,60]. Early examples of IL methods, such as behaviour cloning (BC) (Section 4.1) and learning from observation (LfO) (Section 4), have demonstrated good performance in knot-tying tasks [61].

Speed and quality of simulations have improved tremendously, making it possible to collect millions of data points using simulation for training the perception and control systems and to train a non-linear parametric dynamic model. High-performance simulation also improves the precision of planning/trajectory optimisation methods (Section 5.3) by replacing an analytical model with either the simulation or the learned dynamic model. Most importantly, it makes RL practical in robotic applications by enabling safe and fast online exploration and data collection. Consequently, *simulation-to-reality (Sim2Real)* transfer [62] (Section 3.4) becomes a key technology in modern robotics for bridging the gap between the simulation-trained policy and the real-world deployment.

End-to-end policy learning attempts to eliminate the selection of intermediate state representation from the design process by learning a latent representation so that a control algorithm can be applied to multiple domains. However, neural network parameters must be trained from scratch for different tasks. Hence, transfer learning (TL) is often applied to transfer the domain knowledge learned from a certain task to other domains, where it can be applied directly (zero-shot transfer) or after a few trials (few-shot). TL is also the key technology used in *Sim2Real* (Section 3.4) and Multi-task RL [63]. End-to-end policy learning suffers from data efficiency and mode collapse (similar but different state leads to the same action). Representation learning (Section 5.2) [64] aims to learn better latent state estimation to overcome both issues, leading to robust and interpretable control systems.

DRL and DIL often come with a set of hyper-parameters which have to be manually tuned for each domain. To automate this process, meta-learning attempts to dynamically adjust the hyper-parameters regarding its experience in the target domain and training status [65]. Another hand-designed aspect of many current robotics algorithms is the reward function. Adversarial inverse reinforcement learning (Adversarial IRL) methods [66] also attempt to automate reward engineering with demonstration data while preserving similar computational complexity as their RL counterparts [67,68]. So far, meta-learning methods and Adversarial IRL have not been used in the CDO domain.

### 3.2. Framework

**Markov decision process** (MDP) [69,70] is a mathematical framework that models the decision-making process in a stochastic system and produces corresponding rewards for the decision steps. They have become a strong mathematical tool in many disciplines, including robotics. An MDP M is defined as a 5-tuple $\left(S,A,P,R,\rho_{0}\right)$, where

- S is a set of states;
- A is a set of actions;
- $P_{s,s^{\prime }}^{a}=\mathrm{Pr}\left(s_{t+1}=s^{\prime }|s_{t}=s,a_{t}=a\right)$ is the transition probability function;
- $R_{s,s^{\prime }}^{a}$ is a primary reward function that produces a reward when transitioning from state s to state $s^{\prime }$ by taking action a;
- $\rho_{0}$ is the initial state distribution.

The defining feature of an MDP is that it has the Markov property, i.e., the transition function and the reward function only depend on the current state and action, not past trajectories. This means that while modelling a robotic application, we do not have to consider the effect of state–action sequences; we assume each state is unique and informative enough for transition and decision-making.

**Hidden Markov Model (HMM) [71]** is a first-order Markov process whose internal states S are not directly observable. The observable output signal X depends on probability distributions O. HMM can disregard noise through stochastic framework fitting with the *Expectation-Maximisation (EM)* algorithm [72] and captures the highly stochastic underlying structure of the process. It can model perception systems that provide a prior to the state estimation for a manipulation task [73,74] and detect execution states of RMSs, such as success and various error cases. It is also a framework to formalise trajectory behaviour cloning methods (Section 4.3) that has been used in the dressing domain (Section 6.3.1).

A **Partially Observable MDP [75]** PM combines an MDP and an HMM to model an agent’s decision process where the agent can only partially observe the state of the domain. In addition to the elements of an MDP, a POMDP also contains (X,O) that models the observation space X and emission probability O of the observation x from a state s. POMDPs more accurately describe real-world applications of robotic systems [7] and are a major tool for training modern DRL and DIL end-to-end policy controllers.

**Contextual MDP** (CMDP) [76] is a tuple CM=(Ξ,M), where Ξ is the context space and M is a function that maps a context to a specific MDP $M\left(\xi \right)=\left(S,A,P\left(\xi \right),R\left(\xi \right),\rho_{0}\left(\xi \right)\right)$. In robotics, context captures the across-task variations [47] and it can help to formalise robust and multi-task control settings. We can regard **Goal-conditioned MDP** as a special subset of CMDP, where a goal g∈G only influences the reward function. Skill controllers (Section 3.3) and goal-conditioned RL (Section 5.4) are built upon this framework, and its application will be discussed in cloth-shaping tasks (Section 6.1.2).

### 3.3. Skills

Skills are high-level action abstractions that can be reapplied during different phases of a task and across task families. For example, laundry folding requires applying cloth-grasping and cloth-flattening skills multiple times to perform the long-horizon multi-step task successfully. Some skills require part-based representations, i.e., the presence of a handle on a bag helps an agent know where to grasp the object [48,77]. Other skills relate to the mode switch in the environment, where the actuation of the environment changes in a piecewise fashion [47]. Solving long-horizon multi-step problems usually involves (1) decomposing a task into component skills with regards to the modular structure, (2) developing skill policies independently and (3) using higher-level policy to decide when to use which skill.

In robotics, skills are often modelled using the **Option** framework [78]. Apart from state representation, some skill policies are also subject to the context of the environment. Given a skill library Ω, i.e., a collection of skills, an agent is expected to know when to execute and terminate a certain skill ω, meaning that the agent needs to know the pre-condition and post-condition of executing a certain skill. This supports abstract task-level planning and improves the interpretability of the system [48].

There are two major approaches for integrating skills: (1) segmenting demonstration trajectories into component skills or (2) manually including skill specification as part of the overall problem when learning to solve a task [47], which is mainly used in laundry folding (Section 6.1.1) and knot-tying/untying (Section 6.2.1). Autonomous skill segmentation and discovery [79] could be an interesting direction to explore in CDO manipulation.

Furthermore, transferring the learned skill to another task domain helps the learning efficiency and generalisation ability of the agent. We can initialise a new skill policy with a learned skill policy and fine-tune it in the new task domain [80], which requires a consistent state representation between the two task domains. TL techniques (Section 5.4) such as domain adaptation and domain randomisation [81] are often utilised to build perception systems that produce unified state abstraction for different domains.

### 3.4. Simulation to Reality

Simulation-based approaches provide a cheap and safe development environment for RMSes, but there are challenges to deploying simulation-trained systems into real-world settings [62]. The major challenge is **domain shift**: the simulated MDP differs from the real-world MDP. There is a considerable difference in the observation space and some mismatches in the transition functions. Additionally, there is always a risk that the agent will encounter novel states in the actual trials that it did not encounter in simulation [82].

In classical robotics, the mismatch caused by the dynamic model is partially mitigated through system identification [83] that calibrates the simulator using real-world data. The perception mismatch is resolved with a fixed intermediate state representation between the simulation and the reality. However, since the intermediate state representation is automatically learned in an end-to-end manner in modern robotics, small perturbations in the observation space can cause a significant difference in the latent state representation; consequently, this can lead to a substantial deviation from the correct policy.

TL attempts to improve the performance of a learner on a target domain by transferring the knowledge from a different but related source domain [84]. **Domain adaptation** is maps observations emitted by the same state from the two domains into the same state abstraction. **Domain randomisation** [85] is another TL approach which feeds the agent with observations from a set of randomised domains to fill the observation space gap.

Apart from bridging the gap in the observation space, we also want to transfer the policy from the simulation to reality. In the ideal case, with a robust perception system that gives a consistent intermediate representation and perfectly tuned simulation dynamic, the control system would have no trouble deploying its policy from simulation to reality (zero-shot/direct transfer). However, we cannot guarantee perfect simulation in practice so a simulation-trained agent often runs into novel states in reality. We refer to Zhao et al.’s (2020) review [62] for further details on *Sim2Real*.

### 3.5. Safety

Safety is another major concern in robotic applications, especially in real-world trials. A robot arm needs to avoid jiggering motion and reaching beyond the boundary of its working space to prevent damaging its own body, sensors and motors. It also needs to avoid collisions and exerting excessive forces on objects, humans and collaborators.

With access to a sufficient state representation, these requirements can be met using smoothed motion planning and low-level compliant controllers [86]. Nevertheless, the exploration of RL algorithms presents challenges for training the robot safely in the real world. Although one can use simulators to train the agent in simulation and deploy it to the real world, the robot will typically require extra exploration to adjust to the physical trials, making Safe RL [87] an important topic for modern robotic applications. One approach to Safe RL is adding risk metrics to the reward function and constraints to the action space to penalise the agent for taking destructive actions and reaching unsafe states [31,38]. Another approach is to initialise the policy with a demonstration trajectory and explore around the trajectory to fine-tune its policy [1] (Section 5.5.2). Section 6.3.1 and Section 6.3.2 will discuss the application of conventional safe control strategies and Safe RL in the dressing domain.

## 4. Imitation Learning for CDO Manipulation

Imitation learning (IL) [8] is the first type of data-driven control method that has been used considerably in all four families of the CDO domain (Section 6). Also known as learning from demonstration (LfD) [88], IL learns policy from demonstration as the raw input:

(1) $$\mathrm{IL}\left(\pi^{demo}\right)≐\underset{\pi \in \Pi }{arg min}D\left[p\left(\pi^{demo}\right)\left|\right|p\left(\pi \right)\right]\;\;,$$

where *D* is the divergence metric that measures the difference between the two distributions; the operands in the divergence can be state–action distribution, trajectory feature expectation or trajectory feature distribution. The fundamental difference between IL and RL is that no reward functions are provided in IL, so it helps to eliminate the effort of engineering rewards that can be difficult in many robotics applications.

Behaviour cloning (BC) (Section 4.1) is a type of IL that attempts to directly learn the policy from the demonstration in a way similar to supervised learning. Inverse reinforcement learning (IRL) [89], or inverse optimal control [90], is the second type of IL, which learns the reward function from the demonstration policy and learns the control policy from the inferred reward function. IL can also vary in terms of the control signal given in the demonstration data. The most common IL method applied in CDO is BC, and we are aware of no applications of IRL in the domain.

Demonstration data can be collected through (1) kinesthetic teaching, (2) a motion capture system, (3) a teleoperation system and (4) an expert script policy. Kinesthetic teaching is leveraged frequently in assistive dressing, where a human coordinator grabs the robot arm to demonstrate dressing trajectories, and the data are collected through the robot’s sensorimotors [1]. Motion capture systems collect the demonstration data by keeping track of the movement of a physical expert performing a task. However, such data often present a correspondence problem between the demonstrator and the learner. Teleoperation, on the other hand, requires a human to operate a controller to drive the robot to perform a task. For example, Seita et al. (2019) [21] demonstrate pick-and-place action strategies in vision space to perform bed-making through a click interface. Vinh et al. (2012) [91] employ a joystick to control the gripper’s location and orientation to demonstrate single-gripper overhand knot-tying in the air. Finally, expert-script policies are functional systems that can perform the target task relatively well in real-world or privileged policies that can gain access to the true state of the environment in simulation. The advantage of such a data-collection system is that it frees human demonstration. Such demonstrators have seen substantial use in cloth-shaping systems [21,29,38] and knot-tying systems [92].

Learning from Observation (LfO), or Imitation from Observation, are imitation learning approaches which learn directly from state/observation demonstration data, without action signals. LfO is often applied when the demonstrator’s action space differs from the agent’s. A direct approach is to provide the learner with the keyframes (a sequence of intermediate observations/states) and use lower-level controllers to achieve these goals. For example, Morita et al. (2003) [61] propose *Knot Planning from Observation (KPO)* that integrates control methods from LfO on the topological level of the rope, where the changes of the representation in the consecutive demonstrated observation produce a primitive action. Human imitation learning attempts to infer the intent of the demonstration and probably make a different action than the demonstration—this is analogous to performing LfO using IRL, where the reward function depends only on state/observation signals [93].

Although IL sounds like an ideal approach to tackling robotic applications, with BC as the most prevalent method in practice [8], it still suffers from several issues. The major bottlenecks are demonstration data that can (1) be suboptimal, (2) contain noise and (3) have a different domain (different MDP/POMDP) compared to the application domain [8].

### 4.1. Behaviour Cloning

Behaviour Cloning (BC) [94] achieves IL by learning the policy directly from demonstration data. Concerning policy abstraction, BC can be classified into methods that learn state–action policy, trajectory-level planning and task-level planning. Regarding the involvement of a dynamic model, BC can also be classed into model-free and model-based methods [8].

Model-based BC (MBBC) [95,96] methods are mainly adopted to solve the **corresponding problem** [97] that often appears when the embodiment of the demonstrator differs from the learner. Furthermore, it is hard to apply trajectory-level MFBC to under-actuated systems where the set of reachable states is limited. In contrast, it is possible to plan a feasible trajectory close to the demonstration trajectory in such settings using a dynamic model [98]. As there are no applications of MBBC in the CDO domain, we only focus on the details of MFBC algorithms.

A general algorithmic framework for MFBC is: (1) Collect demonstration data D. (2) Select a policy representation $\hat{\pi }$. (3) Select an objective function L. (4) Optimise L w.r.t. $\hat{\pi }$ using D. A simple approach to BC is to use supervised learning (SL) [99], but this cannot learn to recover from failures during the test time because of the **compounding error**: a cascade of errors from the learned policy. In a supervised approach to BC, we assume that actions in the expert trajectories are independent and identically distributed (i.i.d.) but causal relationships exist in BC applications between the action and the states, which contradicts the i.i.d. assumption. Additionally, SL methods cannot capture other constraints of the robot system, such as joint limits and workspace configuration [100]. Section 6.1.2, Section 6.2.2, Section 6.3.2 and Section 6.4.1 discuss the application of SL methods in CDO manipulation.

### 4.2. State-Action BC

Ross et al. (2011) [101] propose *Dataset Aggregation (DAgger)* to solve compounding error, which can be regarded as a reduction of IL to SL with interaction. *DAgger* maintains one learning policy and iteratively optimises it by collecting aggregated demonstration data. Section 6.1.2 will discuss the application of DAgger in cloth-shaping tasks. *DAgger by Coaching* [102] and *Data as Demonstrator (DaD)* [103] have been proposed to improve learning performance of *DAgger*, but they have not been applied in the CDO domain.

In robotics, Zeng et al. (2021) [104] propose *Transporter Net* to solve vision-based pick-and-place BC using NNs. *Transporter Net* leverages two convolutional networks $Q_{pick}\left(x\right)$ and $Q_{place}\left(\cdot |x,a_{pick}\right)$, where the latter is composed of key and query networks. They output a state–action value map for picking and placing positions in pixel space. The architecture learns to detect the pick action $a_{pick}={arg max}_{x,y}Q_{pick}\left(\left(x,y\right)|x\right)$, and uses the feature map of the region-of-interest of the pick action $x[a_{pick}]$ to cross-correlate to the feature map of the observation x for estimating its place action, i.e., $a_{place}={arg max}_{x,y}f_{query}\left(x\left[a_{pick}\right]\right)*f_{key}\left(x\right)$. The whole inference process is trained end-to-end on the demonstration data. Seita et al. (2021) [34] extend *Transporter Net* to the goal-conditioned control domain and apply it to cloth-shaping and bag-manipulation tasks in simulation. This is discussed in detail in Section 6.1.2 and Section 6.4.1.

### 4.3. Trajectory BC

The simplest form of Trajectory BC is trajectory replay, where the system detects a state of interest and then replays the associated demonstrated trajectory. These methods are only functional under a small state subset of the task domain, and often require extra control to reach and detect these states of interest. Vinh et al. (2012) [91] and Kudoh et al. (2015) [105] apply such methods to achieve knot-tying tasks, which will be addressed in Section 6.2.2.

One direct way to model trajectory learning is using HMM. However, an HMM cannot produce long smooth action sequences as it is a discrete process [8]. Calinon et al. (2010) [106] leverage Gaussian models with HMM to represent continuous values. Yu (2010) [107] proposes *Hidden Semi-Markov Model* (HSMM) to formulate state duration distribution, and Rozo et al. (2016) [108] adopt *Linear Quadratic Regulator (LQR)* [109] to optimise the trajectory generated by an HSMM. The application of the framework in the assistive dressing will be presented in Section 6.3.2.

*Dynamic Movement Primitives (DMP)* [110,111,112,113] are a trajectory behaviour cloning method based on a damped forced harmonic oscillator system instead of MDP. They produce smooth movement to reach the goal within the desired duration:

(2) $$\gamma^{2}\ddot{s}=\alpha_{s}\left(\beta_{s}\left(g-s\right)-\gamma \dot{s}\right)+f\;\;,$$

where $\left(\alpha_{s},\beta_{s}\right)$ are constants that control damping and spring factors individually; γ is a constant that controls temporal behaviour; and the goal configuration g is given by the last state of a demonstration trajectory. The force controller is represented by a weighted combination of basis functions ${\left\{\psi_{i}\right\}}_{i=1}^{N}$ (usually Gaussians). These weights are learned by minimising the sum of squared errors between demonstrated target force and the controller force across time. Section 6.3.2 will discuss the application of *DMP* in assistive dressing.

*DMP* works well for learning point-to-point trajectories as it can easily generalise to different start and goal positions. However, *DMP* generally has limited capability for generalisation and often requires more features for different usage. Gaussian distributions are used in *PoMPs* [114] and *SEDs* [115] for better generalisation behaviour. Schulman et al. (2016) [116], on the other hand, propose to transfer the demonstration trajectory using non-rigid registration between the observed scene and a demonstrated scene. They conduct the registration using the *Thin Plate Spline Robust Point Matching (TPS-RPM)* approach proposed by Chui and Rangarajan (2003) [117] on the scene represented as point clouds. Lee et al. (2015a) [118] extend the work to force control, while Lee et al. (2015b) [119] replace *TPS-RPM* with *Coherent Point Drift* to improve the transferring quality. These approaches eliminate the effort of modelling the distribution over demonstrated trajectories as in *PoDMP* and *SEDS*. The application of *TPS-RPM* has mainly been explored in knot tying/untying tasks, which will be discussed in Section 6.2.2.

## 5. Reinforcement Learning for CDO Manipulation

Reinforcement learning is the second type of data-driven approach used in CDO manipulation. The goal of reinforcement learning (RL) [120], or optimal control [121], is to optimise policy π by maximising expected future accumulative reward, known as return *R* [122]:

(3) $$\mathrm{RL}\left(M\right)≐\underset{\pi \in \Pi }{arg max}\underset{\tau ∼d^{\pi }}{E}\left[\sum_{t=1}^{T}r_{t}\right]\;,\mathrm{or}\;\underset{\tau ∼d^{\pi }}{E}\left[\sum_{t=1}^{T}\gamma^{t-1}r_{t}\right],$$

where τ is a trajectory from the distribution $d^{\pi }$ of trajectories induced by the MDP and policy π; $r_{t}$ represents the reward collected at step *t*, which is equivalent to $R_{s_{t-1},s_{t}}^{a_{t-1}}$—in the case of deterministic dynamic or stochastic dynamic with low uncertainty, we can approximate it with $r(s_{t},a_{t})$; and γ is the discount factor between 0 and 1 to control the importance of a reward with the increment of its collected future step, and it is often adopted in the infinite-horizon case to bound the trajectory return.

RL can be classified into model-free (Section 5.1) and model-based (Section 5.3) RL with respect to the learned/existing dynamic model. RLs can also be categorised in terms of how they use training data: (1) on-policy RL only allows updating its policy based on the trajectories generated by its current policy; (2) off-policy RL trains on the data collected using different policies than the current RL policy, but the agent still explores the environment; (3) offline RL, a special case of an off-policy agent, trains only on pre-collected data. Additionally, maximum-entropy RL (Section 5.5.1) incorporates the *Maximum Causal Entropy Principle* to model humans’ stochastic behaviour and, in turn, produce better exploration policy and robust target policy. Lastly, contemporary RL algorithms, usually DRL algorithms, vary in terms of their representation learning (Section 5.2) and exploration strategies (Section 5.5).

**Exploration** and **reward shaping** are two significant challenges to applying reinforcement learning in practice. With the introduction of NNs, RL algorithms have become practical in high-dimensional, even continuous-state settings. However, this introduces many other new challenges [7]: (1) theoretically, we have **no guarantee of convergence** of the DRL algorithms; (2) **data efficiency** (both sampling and learning efficiency) is a major concern for such complex settings; (3) DRL algorithms often come with a set of **hyper-parameters**, and the algorithms are often sensitive to these hyper-parameters and the application domain; (4) high-dimensional/continuous state settings exacerbate the challenge of **exploration** and further affect data efficiency. The complex **dynamics** of high dimensional/continuous state–action settings further aggravate these challenges.

The application of DRL with POMDP also helps to overcome the challenge of state estimation in robotics [120]. It automates the perception in robotics through end-to-end training and combining multi-modal sensory inputs in the perception network [47]. However, it amplifies the existing challenges in DRL and introduces new ones. First, DRL is data-hungry and trained through trial and error which requires **human involvement** for constantly resetting the environment and preventing unsafe actions [7]. Second, **reward function** needs to be engineered from perception, and this contradicts the idea of tackling the perception in an unsupervised manner. Moroever, even with an oracle (where the agent has access to the true state), reward design is a hard problem in robotics. Third, robotics is usually formulated in a **partially observable** setting, where estimating the true state of the environment is a hard problem. Fourth, RL always deals with **unknown dynamics** of the environment. Last but not least, **asynchronous control**, i.e., the delay between action and the sensory input, violates the formulation of MDP, especially for dynamic control settings.

DRL creates possibilities for creating multi-task/goal-conditioned robots (Section 5.4). Conventionally, robots are only designed to perform a single task with slight variations in context. The generalisability of NNs holds the promise of an agent that can perform different types of tasks across different MDPs. However, the **specification of goals** and **inference of the reward function** for such an agent remain a hard challenges.

### 5.1. Model-Free RL

Model-free reinforcement learning (MFRL) aims to solve the sequential decision-making problem without the knowledge of dynamics. It is mainly classified into value-based methods and policy-gradient methods—the final control policy is either choosing the action that leads to the best state–action value (Section 5.1.1) or directly optimising policy against the RL objective (Section 5.1.2). To apply these two classes of methods into continuous action settings, actor–critic (AC) methods are derived to assist the learning in practice—they learn the parametrised value and policy functions at the same time. Note that some AC methods derive from value-based methods, and some from policy-gradient methods, so we will not discuss AC algorithms separately.

#### 5.1.1. Value-Based Methods

Discrete-action value-based methods *Q-learning* [123] have been applied in cloth shaping (Section 6.1.2) and knot-untying (Section 6.2.2) tasks, while continuous-action methods, such as *DDPG* [124] and *SAC* [125], have mainly been adopted in cloth-shaping tasks (Section 6.1.2). To understand these methods, this section will introduce the evolution of value-based methods from *Q-learning* to *DDPG*.

Value-based methods choose the action that maximises the expected future return at each environment step. Here, we define state $V^{\pi }\left(s\right)$ and state–action $Q^{\pi }\left(s,a\right)$ **value functions** of an MDP (Section 3.2) with their self-consistent **Bellman equations** [120]:

(4) $$\begin{array}{cc} V^{\pi }\left(s\right)=\underset{\tau ∼d^{\pi }}{E}\left[R\left(\tau \right)\;|\;s_{1}=s\right]= & \underset{a∼\pi \left(\cdot |s\right),s^{\prime }∼P\left(\cdot |s,a\right)}{E}[R_{s,s^{\prime }}^{a}+\gamma \;V^{\pi }\left(s^{\prime }\right)] \end{array}$$

(5) $$\begin{array}{cc} Q^{\pi }\left(s,a\right)=\underset{\tau ∼d^{\pi }}{E}\left[R\left(\tau \right)\;|\;s_{1}=s,a_{1}=a\right]= & \underset{s^{\prime }∼P\left(\cdot |s,a\right)}{E}[R_{s,s^{\prime }}^{a}+\gamma \;\underset{a^{\prime }∼\pi \left(\cdot |s^{\prime }\right)}{E}[Q^{\pi }\left(s^{\prime },a^{\prime }\right)]] \end{array}$$

*Q-learning* [123] is an off-policy method that solves tabular MDP problems. It employs an ϵ-greedy behaviour policy to collect online data and a deterministic greedy policy for final testing. The action-state value function is updated using temporal difference (TD) error:

(6) $$Q^{\prime }\left(s_{t},a_{t}\right)=Q\left(s_{t},a_{t}\right)+\alpha \left(y_{t}-Q\left(s_{t},a_{t}\right)\right)\;\;,$$

where the target value $y_{t}=r_{t+1}+\gamma \left(1-d\right)\max_{a_{t+1}}Q\left(s_{t+1},a_{t+1}\right)$ is estimated using the value of the target policy and α is the learning rate.

*Deep Q-learning (DQN)* [126] extends *Q-learning* to solve high-dimensional/continuous state problems in practice, where actions are still discrete. *DQN* leverages a NN to approximate the state–action value function—the network takes true state vectors or observations as input and produces a vector of Q-values for each action. The Q-network is updated using the gradient of the TD error, as shown in Equation (6). Apart from the off-policy strategy used in *Q-learning*, it also utilises a replay buffer to learn from all the historical transitions to improve learning efficiency; however, it can produce a biased estimate of the Q-value. To mitigate the bias, it uses an additional target network, updated periodically from the training network with *polyak averaging* (soft update, where the new target network is the interpolation between the old target network and training network) [6] to estimate the target value, which also helps to stabilise the training. *Double Deep Q-learning (DDQN)* [127] further reduces the bias by choosing the next action using the training Q function and evaluating using the target one.

In order to apply *Q-learning* in a continuous action space, we can obtain the maximum Q-value using policy-network and optimise it with gradient ascent on expected Q-values. This starts to blur the boundary between value-based and actor–critic methods. Such algorithms include *DDPG* [124], *TD3* [128] and *SAC* [125]. These methods use mean squared Bellman error (MSBE) to optimise the critic function:

(7) $$L\left(D\right)=\underset{(s,a,r,s^{\prime },d)∼D}{E}\left[{\left(\hat{Q}\left(s,a\right)-\hat{y}\left(s,s^{\prime },a,d\right)\right)}^{2}\right]\;\;,$$

where the target value is $y\left(s,s^{\prime },a,d\right)=R_{s,s^{\prime }}^{a}+\gamma \left(1-d\right)\max_{a^{\prime }}\hat{Q}\left(s^{\prime },a^{\prime }\right)$.

*Deep Deterministic Policy Gradient (DDPG)* [124] extends DQN by learning a Q-function and a deterministic action network at the same time. It optimises (1) MSBE for training the critic and (2) the expected Q-value of the policy for training the actor. *DDPG* calculates the target value *y* from the target Q-network with the action generated by the current policy. It uses Gaussian action noise to explore the environment.

*DDPG* fails in some scenarios due to overestimating Q-values. *Twin Delayed DDPG (TD3)* [128] mitigates this by introducing clipped *double Q-learning*, delayed policy updates and target policy smoothing. In delayed policy updates, the policy and the target networks are updated less often than the value network. In target policy smoothing, *TD3* augments the target action with noise to prevent the policy from exploiting the value function errors. *Soft Actor-Critic (SAC)* [125] employs the framework of maximum-entropy RL to produce stochastic action behaviour and is discussed in Section 5.5.1.

#### 5.1.2. Policy-Gradient Methods

In CDO manipulation, policy-gradient methods *VPG* [1] and *TRPO* [31,32] are applied in dressing in simulation (Section 6.3.2). This section will introduce the policy-gradient methods from the original *REINFORCE* [129] algorithm to the advanced *PPO* [130] method.

Policy gradient is a direct method to achieve the RL objective (see Equation (3)) by optimising the parametrised policy $\pi_{\theta }$ using its gradient against the objective:

(8) $$\nabla_{\theta }J\left(\pi_{\theta }\right)≅\underset{\tau ∼d^{\pi_{\theta }}}{E}\left[\sum_{t=1}^{T}\nabla_{\theta }\log\pi_{\theta }\left(a_{t}|s_{t}\right)\Phi_{t}\right]\;\;,$$

where $J\left(\pi_{\theta }\right)=\underset{\tau ∼d^{\pi_{\theta }}}{E}\left[\sum_{t=1}^{T}r_{t}\right]$, and $\Phi_{t}$ can be (1) return of a trajectory R(τ)[129], (2) reward-to-go $R_{t}\left(\tau \right)=\sum_{t^{\prime }=t}^{T}r_{t^{\prime }}$, (3) action-value function $Q^{\pi_{\theta }}\left(s_{t},a_{t}\right)$, (4) reward-to-go minus a baseline $b_{t}$ (usually, the value function $V^{\pi_{\theta }}\left(s_{t}\right)$), or (5) the advantage function $A^{\pi_{\theta }}\left(s_{t},a_{t}\right)=Q^{\pi_{\theta }}\left(s_{t},a_{t}\right)-V^{\pi_{\theta }}\left(s_{t}\right)$. In this section, we will talk about policy-gradient algorithms based on these variants.

The choice of $\Phi_{t}$ and the approach to estimating values are key to reducing the variances and biases of the policy gradient. Common techniques include causality trick, baseline subtraction and multi-step estimation. Policy gradient is an on-policy approach due to the distribution of the expectation in the gradient, which introduces biases in the off-policy policy-gradient formulation. Another issue with policy-gradient methods is that small parameter space updates can cause big policy space changes and make training unstable. Variants of these techniques lead to policy-gradient actor–critic algorithms.

*REINFORCE* [129] is the original policy-gradient method for solving RL problems in low-dimensional tabular cases, where true equality holds in Equation (8), i.e., $\Phi_{t}=R\left(\tau \right)$. *REINFORCE* iteratively improves the policy using approximated policy gradient with sampled trajectories and the return of those trajectories:

(9) $$\nabla_{\theta }J\left(\pi_{\theta }\right)\approx \frac{1}{N}\sum_{i=1}^{N}\sum_{t=1}^{T}\nabla_{\theta }\log\pi_{\theta }\left(a_{t,i}|s_{t,i}\right)R\left(\tau_{i}\right)\;\;,$$

where *N* represents numbers of different sampled trajectories. This algorithm does not always work well because the optimisation suffers from high variance in the gradient.

*Vanilla Policy Gradient* (VPG) [129] updates the policy function based on the advantage function which represents the difference between the reward-to-go $R_{t}\left(\tau \right)$ (causality trick) on trajectories generated by the latest policy $\pi_{\theta }$ and parametrised value function $V_{\phi }\left(s_{t}\right)$ as a baseline $b_{t}$, so $\Phi_{t}=R_{t}\left(\tau \right)-V_{\phi }\left(s_{t}\right)$ to reduce the variance in the gradient. The value function is updated using the mean squared error between the value estimation and reward-to-go of all the states on the collected trajectories:

(10) $$\phi^{*}=\underset{\phi }{arg min}\frac{1}{N\times T}\sum_{i=1}^{N}\sum_{t=1}^{T}{\left(V_{\phi }\left(s_{t,i}\right)-R_{t}\left(\tau_{i}\right)\right)}^{2}\;\;,$$

where $\phi^{*}$ represents the parameters of the optimal value function. As VPG trains a stochastic policy, action steps for exploration are sampled from the latest stochastic policy. However, there is a risk of the algorithm converging to local minima, as the policy becomes less random at the end of the training and encourages the agent to exploit previously encountered states. Furthermore, slight changes in the parameter space can strongly affect the policy space. One can use first-order gradient descent to control the updating step on the parameter space, but we want to control the changes in the policy space. *Natural Policy Gradient* (NPG) [131,132] resolves the issue by adding a constraint on the policy space:

(11) $$\begin{array}{cc} \; & \theta^{*}=\underset{\theta^{\prime }}{arg max}{\left(\theta^{\prime }-\theta \right)}^{⊤}\nabla_{\theta }J\left(\pi_{\theta }\right)\\ \; & s.t.\;D[\pi_{\theta^{\prime }}||\pi_{\theta }]\leq \epsilon \;\;, \end{array}$$

where *D* is the divergence measure between two distributions; usually, KL-divergence is chosen: $KL[\pi_{\theta^{\prime }}\left|\right|\pi_{\theta }]\approx \left(\theta^{\prime }-\theta \right)F\left(\theta^{\prime }-\theta \right)$, where *F* is the *Fisher-information* matrix [133]: $F=\underset{\pi_{\theta }}{E}\left[\nabla_{\theta }\log\pi_{\theta }\left(a_{t,i}|s_{t,i}\right)\nabla_{\theta }\log\pi_{\theta }{\left(a_{t,i}|s_{t,i}\right)}^{⊤}\right]$.

*Trust Region Policy Optimisation (TRPO)* [134] attempts to make the most significant possible improvement to policy parameters without causing performance collapse. It uses surrogate advantage that measures how policy $\pi_{\theta }$ performs compared to another policy $\pi_{\theta^{\prime }}$. *TRPO* updates its policy using the surrogate advantage while keeping a constraint that limits how different the new and old policies are allowed to be to avoid taking a big step:

(12) $$\begin{array}{cc} \; & \theta_{new}=\underset{\theta }{arg max}\underset{a∼\pi_{\theta },s∼P}{E}\left[\frac{\pi_{\theta }\left(a|s\right)}{\pi_{\theta_{old}}\left(a|s\right)}A^{\pi_{\theta_{old}}}\left(a,s\right)\right]\\ \; & s.t.\;\underset{s∼\rho_{\pi_{\theta }}}{E}\left[KL\left[\pi_{\theta }\left(\cdot |s\right)\left|\right|\pi_{\theta_{old}}\left(\cdot |s\right)\right]\right]\leq \epsilon \;\;. \end{array}$$

Solving this objective is difficult in practice, so *TRPO* approximates the surrogate advantage and the mean of the KL-divergence using the Taylor series up to the first order and second order of the corresponding functions. The objective can be solved by optimising a quadratic gradient that resembles *NPG*.

*TRPO* is conceptually and computationally challenging mainly due to the constraint in Equation (12). *Proximal Policy Optimisation (PPO)* [130] proposes a simpler objective:

(13) $$\begin{array}{cc} \theta_{new} & =\underset{\theta }{arg max}\underset{a∼\pi_{\theta },s∼P}{E}\left[L(s,a,\theta_{\mathrm{old}},\theta )\right]\\ L(s,a,\theta^{\prime },\theta ) & =\min\left(\frac{\pi_{\theta }\left(a|s\right)}{\pi_{\theta^{\prime }}\left(a|s\right)}A^{\pi_{\theta^{\prime }}}\left(a,s\right),\;\mathrm{clip}\left(\frac{\pi_{\theta }\left(a|s\right)}{\pi_{\theta^{\prime }}\left(a|s\right)},\;1-\epsilon ,\;1+\epsilon \right)A^{\pi_{\theta^{\prime }}}\left(a,s\right)\right)\;\;, \end{array}$$

where several steps of gradient ascent can deliver the policy update. Clipping is a regulariser that makes changes in policy less drastic, but it can still allow large step updates to the policy. There are many techniques to resolve this issue, but the update can be cancelled if the original constraint goes beyond a certain threshold. *PPO-Penalty*, on the other hand, subtracts the KL-divergence from the objective as a Lagrangian term.

### 5.2. Representation Learning

When developing an end-to-end DRL controller, the quality of the latent representation directly affects the performance of the policy. The aim of representation learning in RL (RLRL) is to learn a good latent representation z from observations x or states s so that it can closely approximate the true state s of a POMDP/MDP. This helps to reduce the *curse of dimensionality* in MDP policy learning and, most importantly, plays the role of state estimation in POMDP settings. Representation learning also helps to improve the **learning and sampling efficiency** of the RL algorithm, as learning policies from state-based input are significantly more sample-efficient than learning from observation [135,136].

Representation learning in DRL builds heavily on work from the wider DL community. It has been argued that a good representation should be able to (1) learn task-relevant information [137], (2) preserve most information to predict the future [138], and/or (3) capture the posterior distribution of the underlying explanatory factors for the observation [139]. Common representation learning techniques used in DRL [137] include (i) data augmentation [64,136,140,141,142] (Section 5.2.1), (ii) policy/value regularisation [140] (Section 5.2.1), (iii) contrastive learning [64,136] (Section 5.2.1), (iv) input/latent mutual information [143], (v) maximising posterior distribution with dynamic [144,145,146,147,148] (Section 5.3.2) or without dynamic learning [149] (Section 5.2.2), (vi) predictive information [138,150,151], (vii) bisimulation [137,152] and (viii) asymmetric training by taking advantage of simulation [153]. So far, only data augmentation, contrastive learning and latent dynamic learning approaches have been applied in the cloth-shaping domain (Section 6.1.2).

The main challenge for representation learning in POMDP settings is to obtain accurate state estimation from partial observation. *DQN* [126] uses a "windowing" technique to concatenate small sequences of observations. *Never Give Up (NGU)* [154] and *Agent 57* [155] learn latent representation using recurrent neural layers to aggregate historical observations. *PlaNet* [156], *Dreamer* [144,145], *SLAC* [157] and *Muzero* [158] further refine the state estimation by combining the prior estimation from the model and posterior estimation from the observations using a dynamic model, which works like a non-linear Kalman filter.

Shelhamer et al. (2016) suggest that DRL algorithms should spend considerable time on representation learning [159]. This encourages pre-training of the representation before training the exploratory RL agent. They can be trained with RL objectives simultaneously in an end-to-end fashion or different parts of the agent can be trained in an alternating fashion throughout online training. Representation learning techniques can be applied to offline data to train a good observation/state encoder E in an unsupervised and/or self-supervised way before interacting with the environment.

#### 5.2.1. Data Augmentation

Data augmentation (DA), such as random flipping and cropping of images, is a type of upsampling and regularisation method that has demonstrated the ability to improve data efficiency and generalisability of supervised computer vision tasks. In general, DRL encoders suffer from observation overfitting [160,161], so DA can also be used to improve **data efficiency** and **generalisation** ability of a DRL agent [141,142]. It has been shown that *Random Shift* can overcome the overfitting problem of vision encoders [140]. We refer to Laskin et al. (2020) [141] for a comparison of available DA functions in the DRL domain—they experimentally show that *Random Crop* provides the best performance improvement.

DA improves the generalisation ability of the agent by letting it learn to extract observation features that matter to the task performance. It can be treated as a domain randomisation technique that adds variations to the observation space. However, optimisation of RL becomes increasingly challenging with the increasing variation in the observation due to the limited expressiveness of NNs [142]. It has been shown that DRL using DA can suffer from unstable training and poor performance [141].

DA is often applied with contrastive representation learning that further improves the data efficiency of downstream tasks by learning good latent representation [162,163,164]. As a sub-discipline of self-supervised learning, *contrastive learning* (CL) aims to create an embedding space where similar samples remain close while dissimilar ones are far apart. CL achieves its goal by automatically labelling positive and negative pairs and optimising a contrastive loss function. It has been successful in computer vision, natural language processing and reinforcement learning [136,165].

*Contrastive Unsupervised Representations for Reinforcement Learning (Curl)* [136] utilises CL on the visual observations with DA to produce a better latent representation of the state for the RL algorithms. *Soft Data Augmentation (SODA)* [142] further improves **sample efficiency** and **stabilises** the RL optimisation by decoupling data augmentation from policy learning. It adds a soft constraint that maximises the mutual information between the augmented and non-augmented data, while underlying RL uses non-augmented data. Experiments suggest that *Random Overlay* [137] (which interpolates the observation with an unrelated image and achieves *Bisimulation* in practice) is the best DA technique to improve data efficiency of pixel-based DRL.

Augmented observations can also be used to regularise the RL objective function. *Data-regularised Q (DrQ)* [140] leverages both data augmentation and explicit regularisation term that is added to the value-based RL objective. Since DA introduces variations in observation space, value functions become unstable to train, although DRL ultimately benefits from it. An explicit regularisation term is used to stabilise the training. Generally, it follows three steps: (1) Data augmentation with small random shifts; (2) Average target Q-value over several image transformations; (3) Average Q-value estimation over several data transformations. *Curl* and *DrQ* have been examined in cloth-shaping tasks in simulatioan [29], which will be discussed in Section 6.1.2.

#### 5.2.2. Posterior Latent Distribution

Before introducing the RLRL techniques that maximise the posterior latent distribution, we briefly cover the variational autoencoder which is the common tool used to achieve such objectives. *Variational autoencoder (VAE)* learns the latent feature distribution of the data. It is an implementation of *Amortised Variational Inference* that approximates the posterior p(z|x) with a stochastic inference function $q_{\phi }\left(z|x\right)$[166]. *VAE* can also be treated as a generative model (GM) trained with variational inference; it has a generative model $p_{\theta }\left(x|z\right)$ that can generate new data points from a latent distribution:

(14) $$\mathrm{GM}\left(D,x\right)≐\underset{\theta }{arg min}D\left[p_{D}\left(x\right)\left|\right|p_{\theta }\left(x\right)\right]\;\;,$$

where D represents the dataset and D[·||·] is a divergence metric. Expanding the objective with KL-divergence, the optimisation is equivalent to maximising the expected marginal log-likelihood of the observable variables over the data distribution:

(15) $${\mathrm{GM}}_{KL}\left(D,x\right)≐\underset{\theta }{arg max}\underset{p_{D}\left(x\right)}{E}\left[\log p_{\theta }\left(x\right)\right]\;\;.$$

In the case of *VAE*, the marginal log-likelihood term can be optimised by maximising the *Evidence Lower Bound (ELBO)*:

(16) $$\log p_{\theta }\left(x\right)\geq \underset{z∼q_{\phi }\left(z|x\right)}{E}\left[\log p_{\theta }\left(x|z\right)\right]-KL\left[q_{\phi }\left(z|x\right)\left|\right|p\left(z\right)\right]≐L_{VAE-ELBO}\left(x,\phi ,\theta \right)\;\;.$$

For simplicity, a normal distribution is often chosen for the prior distribution p(z), so that the inference model $q_{\phi }\left(z|x\right)$ infers the means $\mu_{\phi }\left(x\right)$ and standard deviations $\sigma_{\phi }\left(x\right)$ for the latent variational distribution. Subsequently, the objective for *VAE* becomes the following:

(17) $$\begin{array}{c} L_{VAE-ELBO}\left(\phi ,\theta \right)=\underset{p_{D}\left(x\right)}{E}[-\underset{\epsilon ∼N(0,I)}{E}\left[\frac{1}{2\sigma^{2}}\left|\right|p_{\theta }\left(e_{\phi }\left(x,\epsilon \right)\right)-x{\left|\right|}_{2}^{2}\right]\\ -\underset{KL\left[q_{\phi }\left(z|x\right)\left|\right|p\left(z\right)\right]}{\underset{︸}{\frac{1}{2}\left(\left|\right|\mu_{\phi }{\left(x\right)||}_{2}^{2}+\left|\right|\sigma_{\phi }\left(x\right){\left|\right|}_{2}^{2}-d-2<\log\sigma_{\phi }\left(x\right),1>\right]}} \end{array}$$

where the latent state z is sampled with *Monte Carlo estimation* with *reparameterisation trick*$e_{\phi }\left(x,\epsilon \right)=\mu_{\phi }\left(x\right)+\sigma_{\phi }⊙\epsilon ,\;\;\epsilon ∼N\left(0,I\right)$.

*VAE* is an important tool used in many RLRL approaches for learning a maximum posterior latent distribution. Such posterior latent distribution can be better estimated with a latent dynamic model, such as *Recurrent State Space Model* [156] (Section 5.3.2) and *Control as Inference* in POMDP [147] (Section 5.5.1).

### 5.3. Model-Based RL

Model-based reinforcement learning (MBRL) [167] can be subdivided into three main types: those that **plan using a learned model/known model** (Type I), **learn policy/value with imagined trajectory induced by learned model** (Type II), or **implicitly learn the transitional structure of the domain** (Type III). MBRL is generally more data-efficient than MFRL algorithms and it generalises better with large and diverse data [7]. Type I MBRL is commonly applied in cloth shaping (Section 6.1.2) and assistive dressing (Section 6.3.2), while we are aware of no application of Type II and III MBRL in CDO.

Planning, or trajectory optimisation, algorithms [121] usually generate local solutions for a subset of the state space. They often need to gain access to the dynamic model of the environment, i.e., either a known dynamic P or a learned dynamic $\hat{P}$. Common planning algorithms in DRL domains include *Model Predictive Control (MPC)* [168], *Linear Quadratic Regulator (LQR)* [109] and *Monte-Carlo Tree Search (MCTS)* [169]. We refer readers to the review by Moerland et al. (2020) [170] for a more detailed survey of planning algorithms.

To avoid risky and erroneous actions caused by the **compounding error** in the model, we can choose the first action generated from the trajectory from the policy $\pi_{\hat{P}}$. Then, we **replan** for other future actions. The most direct way to reduce the compounding error in planning is to (1) use **multi-step prediction** in model training [171,172] and (2) have a **short planning horizon**. In addition, epistemic uncertainty is partially caused by **overfitting** the model to the training data. **Model exploitation** [7] refers to planning methods with learned models that can exploit the epistemic uncertainty in training and testing time. In addition to techniques discussed above, another way to mitigate the issue is to incorporate the **uncertainty estimation** for policy generation.

In the case of an unknown or expensive dynamic, the simplest way to learn an approximated dynamic model $\hat{P}$ is to train it using SL [173]. Transitional samples can be induced by a **base policy**$\pi^{base}$, such as a random policy. This approach generally does not work well in practice, especially in high-dimensional settings [37,38], mainly due to the **distributional shift** problem, i.e., $\rho_{\pi_{\hat{P}}}\left(s_{t}\right)\neq \rho_{\pi^{base}}\left(s_{t}\right)$[8]; the issue is exacerbated when an expressive model is adopted. Distributional shift can be partially mitigated by iteratively gathering samples from the policy $\pi_{\hat{P}}$ and training a new model ${\hat{P}}^{\prime }$ using the **aggregated dataset** as in BC (Section 4.1). In training, it is better to collect transitions that lead to expected high rewards under the uncertainty of the dynamic [7]. We can also utilise **demonstration data** to further reduce the distribution shift, as it causes the model to learn the important part of the state space [7].

#### 5.3.1. Observational Dynamic Models

The ability to predict future outcomes of actions for a given event is a key component of human decision making. The human brain has a complex embedded representation of physical and causal rules of the world [174]. Such a predictive ability lets an RL agent plan either based on observations or the latent space. Such a model can also provide a good latent representation for policy learning, which helps improve **data efficiency**. Agents can further benefit from the observational dynamic model by learning from imagined trajectories. However, learning such a dynamic observational model is difficult for an RL agent due to the **aleatoric uncertainty** and **partial observability** of the environment.

In high-dimensional settings, *Variational Inference* (Section 5.2.2) can be used with an NN-parameterised dynamic model to account for the aleatoric uncertainty in the system [156]. Partial observability is another factor that accounts for epistemic uncertainty. Attempts to leverage recurrent state–action transitions for better representation learning and state estimation include *RSSM*-based algorithms [156,156] and *Visual MPC* algorithms [175].

The most popular approach in such settings is anticipating vision outcomes since vision captures rich information about spatiotemporal interactions between objects. Ebert et al. (2018) [175] discuss the important roles of the visual dynamic model, i.e., action-conditional video prediction model, in the POMDP setting of robot control and propose a framework called *Visual MPC* that performs planning on the predicted pixels of the future states. *Visual MPC* using *SV2P* [176] and *SVG* [177] has been investigated in the cloth-shaping domain (Section 6.1.2). Visual dynamic learning becomes more challenging when there are **occlusions** occurring among objects; memory-based approaches, such as recurrent networks, must be adopted in such settings. Good encoding representation from videos is also challenging due to the high dimensionality and variability of the visual space.

#### 5.3.2. Latent Dynamic Models

**Figure 2:**
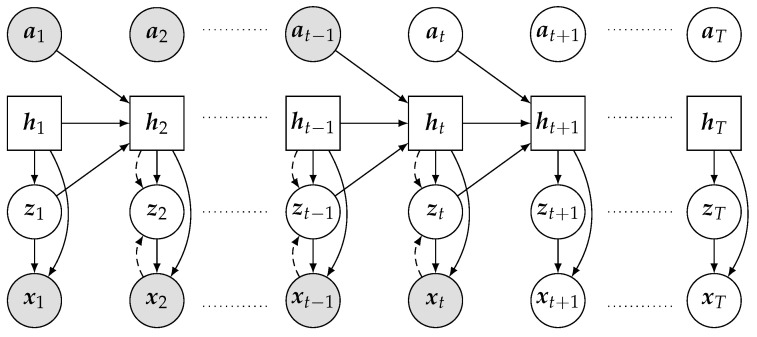
Probabilistic graphical model of the Recurrent State-Space Model (*RSSM*) [147].

Numerous latent dynamic models use vision prediction for representation learning in the RL community. Hafner et al. (2019) [156] propose the *Recurrent State Space Model* (RSSM), which is a probabilistic model that predicts latent states of an environment with a latent dynamic model and refines its prediction based on a sequence of observations. Figure 2 shows the *Probabilistic Graphical Model* (PGM) for the *RSSM* model. As the actual state of the environment s is often inaccessible, *RSSM* is defined in the POMDP setting with the following latent state dynamic: (1) recurrent dynamic model $h_{t}=f\left(h_{t-1},z_{t-1},a_{t-1}\right)$, (2) representation model ${\hat{z}}_{t}∼q\left(\hat{z}|h_{t},x_{t}\right)$ and (3) transition predictor ${\tilde{z}}_{t}∼p\left(\tilde{z}|h_{t}\right)$, where h represents the deterministic latent representation of state s, $\tilde{z}$ represents the prior of the stochastic latent state and $\hat{z}$ is the posterior induced by the deterministic latent state h and the current observation x.

Built upon *RSSM*, *PlaNet* [156] aims to learn the latent dynamic that can generate accurate vision and rewards from a prior latent distribution for *MPC with Cross-Entropy Method* planning, which unrolls and maximises the accumulative future rewards from the latent dynamic distribution. This is achieved by maximising the *ELBO* between prior and posterior latent states and the maximum likelihood of reconstruction of the observation and reward. Thus, it also includes observation predictor ${\hat{x}}_{t}∼p\left(x|h_{t},z_{t}\right)$ and reward predictor ${\hat{r}}_{t}∼p\left(r|h_{t},z_{t}\right)$. Thus, while conditioning on the action, the objective becomes:

(18) $$\begin{array}{cc} L_{PlaNet-ELBO} & =\sum_{t=1}^{T}(-\underset{q(z_{t}|x_{1:t},a_{1:t})}{E}\left[\log p(x_{t}|z_{t})\right]\\ \; & +\underset{q(z_{t-1}|x_{1:t-1},a_{1:t-1})}{E}\left[KL\left[q\left(z_{t}|x_{1:t},a_{1:t}\right)||p(z_{t}|z_{t-1},a_{t-1}\right]\right])\;\;. \end{array}$$

*PlaNet* employs *Gated Recurrent Units (GRU)* [178] as the backbone of the latent dynamic model. It uses MSE as the loss function to learn observation reconstruction and reward prediction, and it adopts a diagonal Gaussian distribution to model the variational variable. To reduce the prediction error, it also utilises multi-step loss, called *overshooting*, on the variational distribution and the reward prediction.

*PlaNet* has been applied in the cloth-shaping literature (Section 6.1.2), but it has so far shown poor performance, likely due to the blurry observation prediction [38,179]. We are not aware of any applications of improved algorithms based on *PlaNet*, such as *Dreamer* [144], *SLAC* [147] and *APV* [148], in any of the CDO domains.

### 5.4. Goal-Conditioned RL

Broadly speaking, multi-task reinforcement learning (MTRL) [63,180,181] refers to a general RL agent that can perform tasks in different domains. In more narrow terms, MTRL is equivalent to **goal-conditioned reinforcement learning** (GCRL), where the agent can perform a given objective across tasks within a dynamic-invariant task family. GCRL, especially *HER* [182], has been extensively applied to cloth shaping (Section 6.1.2).

In GCRL, the goal works as a contextual parameter that only alters the reward function for each MDP (Section 3.2). Goal-conditioned reward functions are often negatively related to the distance between the current state and the goal for training the agent in a self-supervised manner [182,183,184,185,186,187,188]. Such formulation leads to universal value functions [189], also known as goal-conditioned value functions. In robotics, GCRL is the tool to solve robust skills policy. More interestingly, it can be used to improve the sampling efficiency of an RL agent [190] by associating with the idea of deep exploration [191] (Section 5.5).

Andrychowicz et al. (2017) [182] propose *Hindsight Experience Replay (HER)* which trains a goal-conditioned Q-function based on *DQN* and a goal-conditioned policy function based on *DDPG*. This algorithm can be applied to off-policy RL algorithms on multi-goal/single-goal sparse-reward task settings. In *HER*, the replay buffer stores transitional data with the original goal of an episode but also with some modified goals. It was shown experimentally that *HER* results in vast improvement compared with its base algorithm in many hard environments. The challenging part of this algorithm is recalculating the sparse-reward function for the newly sampled goals. It means that the true state of the steps in the latest episode has to be stored temporarily unless the reward can be calculated based on the observations.

### 5.5. Exploration

One of the most important components of an RL agent is its **exploration** strategy of the **behaviour policy**: the policy the agent used to collect transitional data during training. If the agent explores insufficiently, it may not find highly rewarded states, and its policy will be suboptimal. If it finds highly rewarded trajectories but keeps exploiting them, it may also converge to a suboptimal solution because it may miss exploring better trajectories [192]. The balance between exploration and exploitation directly influences the sampling efficiency of an algorithm that, in turn, affects (1) learning efficiency, (2) the upper bound of its performance and (3) the training stability. A better exploration strategy also leads to robust behaviour, as it has undergone various states throughout the training. Exploration is a huge field in RL literature, and we refer readers to related reviews [193,194,195].

Without considering the future outcome in a long horizon task, a good myopic exploration strategy is one that (1) explores states that might lead to better reward gains, known as optimistic in the face of uncertainty, and/or (2) explore states that are novel and provide more information on the environment, known as intrinsic motivation. These two may be dual problems, but with the latter, the agent can explore the environment independently without an extrinsic reward signal. Some fundamental myopic exploration strategies are derived from the *Multi-armed Bandit* (MAB) formulation [196], then scaled to more complex MDP/POMDP settings. MAB-derived strategies focus more on the information about the action, and they can be classified into greedy soft policy, *Upper Confidence Bound* (UCB) [197] and *Thompson Sampling* (TS) [198] approaches.

The DRL community has focused on optimistic methods, including intrinsic motivation [199,200], MaxEnt-RL (Section 5.5.1) and sampling-based methods [190,201,202] based on TS for tackling exploration in complex MDP/POMDP settings. In addition, demonstration learning, i.e., fine-tuning the RL policy combined with BC, is another way to reach asymptotic performance with a small amount of data. Other attempts include injecting parameter noise [203], adversarial self-play [204] and state marginal matching [205,206].

However, there are many issues regarding myopic explorations. In more complex settings (high-dimensional or continuous MDP/POMDPs), simple exploration approaches suffer from large state–action space and from sparse, delayed and deceptive rewards [207], where the latter case is known as the hard-exploration problem [208]. Apart from being unable to explore every possible state–action pair, such long-horizon MDPs have causal structures: some states will not appear unless the agent takes specific trajectories. Additionally, the simple strategy that only explores the surrounding of the policy output with adding noise will often lead to local optima in a continuous action setting. POMDPs introduce another layer of challenges related to accurate estimation of the environment state—higher-quality representations can lead to better exploration [209,210].

Ecoffet et al. (2019) [208] point out that the two direct consequences of myopic approaches are (1) the agent always forgets the highly rewarded region in the state space (**detachment**) and (2) the agent cannot robustly return to such states if remembered (**derailment**). Solving these problems requires a good global exploration strategy [211] that can better balance long-term and short-term environment information [195]. In the literature, such global exploration is also known as **deep exploration** [190]. The deep exploration method considers both myopic information gain and the long-term causal outcome of an action for future learning. An approach to achieving deep exploration is to use goal-based strategies [212,213], where the important states are proposed to guide the exploration of the agent. These approaches need a heuristic goal generator and a lower-level exploration strategy for exploring to achieve the goals and conduct further exploration once a goal is achieved [214]. However, the exploration that focuses on tackling deep exploration has not been utilised in CDO literature.

#### 5.5.1. Maximum Entropy RL

Human behaviour is not optimal most of the time: the trajectory from the starting and end states can vary around the optimal trajectory. Instead of learning deterministic policy, maximum entropy reinforcement learning (MaxEnt-RL) [215,216] incorporates the *Maximum Entropy Principle* to learn a distribution of policies whose mean is close to the optimal policy. The objective is defined as

(19) $$\mathrm{MaxEnt}-\mathrm{RL}\left(M,\alpha \right)≐\pi_{soft,\alpha }^{*}=\underset{\pi \in \Pi }{arg max}\sum_{t=1}^{T}\underset{\left(s_{t},a_{t}\right)∼\rho_{\pi }}{E}\left[r\left(s_{t},a_{t}\right)+\alpha H\left(\pi \left(\cdot \;|\;s_{t}\right)\right)\right],$$

where α is the temperature scalar to interpolate between the original RL objective and MaxEnt-RL Objective. This objective can be understood as optimising the RL objective under the augmented reward function $r_{soft,\alpha }^{+}\left(s,a;\pi \right)=r\left(s,a\right)-\alpha \times KL\left[\pi \left(s\right)||U\right]$, where the entropy of a stochastic policy provides the intrinsic reward.

By introducing binary optimality variables in high-dimensional continuous MDP settings, Levine (2018) [216] shows that MaxEnt-RL can be derived with control as probabilistic inference with known deterministic dynamics in tabular cases and variational inference without the knowledge of dynamics. This led to the MFRL method soft Q-learning [217] and the actor–critic method *Soft Actor-Critic (SAC)* [125].

In POMDP settings, *Stochastic Latent Actor-Critic (SLAC)* [147] improves upon *SAC*’s objective by incorporating latent dynamic learning to learn soft value and policy functions efficiently. These algorithms achieve asymptotic performance using end-to-end learning. *SAC* has been investigated in the cloth-shaping domain (as described in Section 6.1.2), but we are not aware of the application of *SLAC* in the CDO domain.

#### 5.5.2. Demonstration Learning

The most practical way to bypass sample efficiency problems in complex MPD/POMDP is to use demonstration trajectories. These approaches usually use BC methods (Section 4.1) to initialise the policy with demonstration data and leverage online collected trajectories to fine-tune its policy with the RL objective. Demonstration learning (DemoL) algorithms often used in robotics applications are *DQfD* [218], *DDPGfD* [219] and *Q-filtered BC* [220]. In trajectory-level DemoL, a trajectory generated by trajectory BC controllers (Section 4.3), such as *DMP*, is usually refined by trajectory optimisation algorithms such as *LQR* and *Policy Improvement with Path Integrals (PI2)* [221]. In CDO manipulation, DemoL algorithms are mainly used in cloth shaping (Section 6.1.3) to reduce the exploration complexity and in assistive dressing for safe trajectory control tasks (Section 6.3.2).

Building upon *Prioritised Duelling Double DQN* [222], *Deep Q-Learning from Demonstrations (DQfD)* [218] is pre-trained only on the demonstration data using TD (see Equation (6)) and supervised losses. The losses include a one-step double Q-learning loss, a multi-step double Q-learning loss (similar to *A3C*), a supervised large margin classification loss and L2 regularisation on the network’s parameters. Note that these losses are also applied in the second phase of the training on the demonstration data. The supervised loss function [223] is defined as:

(20) $$\begin{array}{cc} L_{DQfD-SL}\left(\hat{Q},a_{demo}\right) & =\max_{a\in A}\hat{Q}\left(s,a\right)+l\left(a_{\mathrm{demo}},a\right)-\hat{Q}\left(s,a_{\mathrm{demo}}\right)\;\;, \end{array}$$

where $l(a_{\mathrm{demo}},a)$ is a margin function; it is 0 when $a_{\mathrm{demo}}=a$, otherwise, it is a positive constant ϵ; it makes the Q-value of the other actions at least a margin inferior to that of the demonstrated one. The agent starts online learning with its pre-trained policy and updates its value function with a mixture of demonstration and collected data. Demonstration data are preserved throughout the training, and a bonus is added to the weights of the demonstration data to increase the possibility that the replay buffer will sample them.

*DQfN* has outperformed its pure RL and IL counterparts on most of the Atari games. As with *DQN* (Section 5.1.1), this algorithm can only be applied to the discrete-action domain. *DDPG from Demonstrations (DDPGfD)* [219] is proposed for continuous cases and can also deal with sparse rewards. The priority of transition data in the replay buffer comprises its latest TD error, actor loss and bonus factor for demonstration data. It uses a mixture of one-step and multi-step loss to train the critic in the sparse reward setting.

Building upon *DDPG* [124] and *HER* [182], Nair et al. (2018) [220] propose a method called *Q-filtered Behaviour Cloning (QfBC)* that also applies the BC loss to learn demonstration data to solve long-horizon, sparse-reward and goal-conditioned tasks. Nevertheless, this method retains two different replay buffers instead: one is for the demonstration data and another for the trial data—it does not utilise priority replay buffers like in *DDPGfD*. At each update step, the algorithm samples a certain amount of data from both replay buffers. The update losses are similar to *DDPGfD* with the difference that it also uses Q-filtered BC loss on the demonstration samples for updating the actor:

(21) $$L_{QfBC}=\sum_{i}\left|\right|\pi_{\theta }\left(s_{i}\right)-a_{i}{\left|\right|}^{2}\;\left[Q\left(s_{i},a_{i}\right)>Q\left(s_{i},\pi_{\theta }\left(s_{i}\right)\right)\right]\;\;,$$

where [·] is a Boolean expression function which equals 1 when its operand is true and 0 otherwise. They also reset some trial episodes using the intermediate states and goals from demonstration episodes. The initial states of the resets are sampled from any intermediate states in a demonstration episode. As *HER* is also utilised, the goal state for these reset episodes is chosen to be the goal state of the same demonstration episode.

## 6. CDO-Manipulation Tasks and Systems

**Figure 3:**
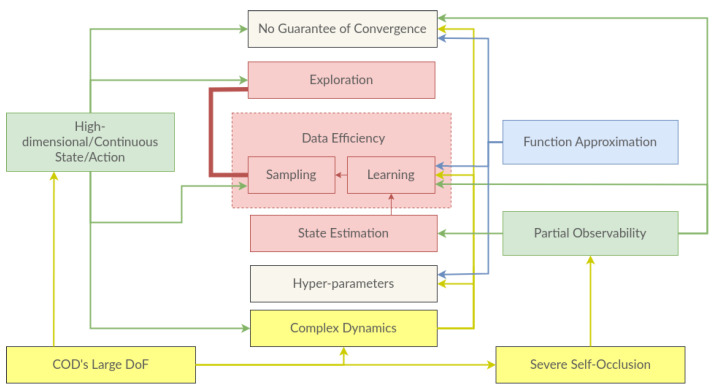
The key challenges of data-driven control in CDO: (in yellow) challenges caused by the nature of CDO, (in green) challenges caused by use of POMDP, (in blue) challenges caused by using neural networks and (in red) key challenges.

The most fundamental challenge in manipulating CDOs is that they have many DoF, which leads to complex dynamics and severe self-occlusion. A shown in Figure 3, these challenges both directly and indirectly affect the different parts of a data-driven method. The complex dynamic of a CDO introduces a major obstacle for manipulation because different parts of the cloth move differently with respect to their individual internal forces. There are aleatoric uncertainties about the cloth’s deformation, meaning that the cloth does not always deform the same way under the same action in the real world. The complex dynamic of CDOs makes analytical model building, dynamic learning and exploration in RL algorithms difficult. Self-occlusion can hide effective key points for manipulation, which makes it hard to extract the state of the cloth and presents challenges for engineering reward functions and automating the goal evaluation for complex CDO manipulation tasks. Another major challenge is grasping. A two-fingered gripper is sufficient for most of the tasks in this domain. Nevertheless, roller end-effectors are also utilised for effective and safe grasping, and multi-fingered grippers are adopted for more dexterous manipulation such as tying a bowline knot [224].

Humans perform different CDO tasks in different ways. In cloth-shaping tasks, we tend to focus on effective points, such as the edges and corners of the article. If an effective grasping point is hidden, we have good intuition to unravel the cloth to find these points. At the end of the task, we can smooth the wrinkles on the cloth by spreading our hands and stretching the corners in the opposite direction. In rope manipulation such as insertion, knot tying and untying, we have the foreknowledge to know where to grasp along the rope. We depend on our vision system for insertion tasks, and we can only depend on haptic sensors and motor skills to finish rope tying and untying tasks. As for bag manipulation, we can detect the hem of the bag for grasping effective points to open it. While lifting it, we can estimate the amount of force we need to hold the bag in our hands without tearing it. In dressing tasks, we usually ignore the deformation of the garment and mainly focus on whether our limbs go into the correct openings of the garments. We mainly rely on our haptic system to sense if we are performing the task correctly and safely.

Table 1 shows the data-drive literature in the four prevailing task families in the CDO domain: cloth-shaping, knot tying/untying, dressing and bag manipulation. In this section, we discuss the individual challenges of these tasks and how the perception and control systems in the literature manage to tackle these challenges.

### 6.1. Cloth Shaping

Cloth shaping is a crucial skill for doing laundry in daily life [251]. We broadly define cloth shaping as manipulating a single CDO, such as a rope, a towel or a T-shirt, to a goal configuration. Narrowly, we define cloth as a square fabric made of any cloth-like material. A canonical task in cloth shaping is cloth flattening, where one or more end-effectors apply actions on a piece of square fabric to unfold it completely on a table.

**Figure 4:**
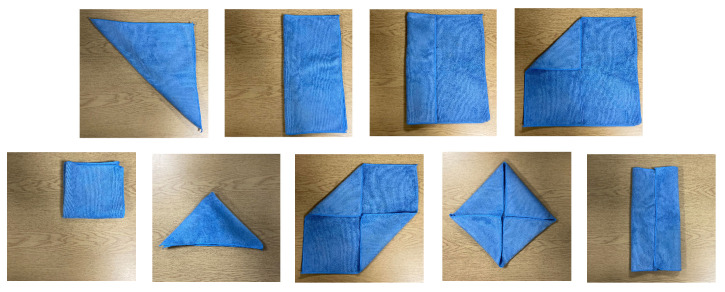
Different folding tasks on a square fabric, where the figure is modelled after Weng et al. (2022) [237]: The first row displays the goal images of single-step folding tasks including diagonal folding, rectangular folding, side folding, one-corner inward folding from left to right; the second shows the ones of multi-step folding, including cross folding, diagonal cross-folding, double-corner inward folding, all-corner inward folding and double side-folding.

A more complex task in cloth shaping is cloth folding, which includes single-step folding (SSCF) and multi-step folding tasks (MSCF), as shown in Figure 4. SSCF refers to folding tasks achievable with one pick-and-place (P&P) action by a human, while MSCF refers to tasks requiring multiple P&P actions [38,237]. In terms of the initial state, folding tasks can also be classified into folding from the flattened shape and folding from the crumpled form (FCF), where the latter is a more complex problem [29].

Flattening, folding, or other types of complex shaping can also be applied to other CDOs [237,252], such as towels (rectangular fabric instead of square), T-shirts, trousers, shorts, dresses, coats and bags. The more complex the shape of the CDO, the harder the task will be. Many tasks in the cloth-shaping domain comprise several subtasks. For example, the system must flatten the object before the actual folding to perform FCF. Similarly, Weng et al. (2022) [237] assign intermediate goals to perform MSCF tasks successfully. Furthermore, the nature of the task depends on the number of end-effectors. Usually, more end-effectors lead to more efficient manipulation. However, we need to consider extra constraints to avoid collisions among the arms. For this reason, comparison between methods using different numbers of end-effectors can be unfair.

#### 6.1.1. Classical Control and Perception in Cloth Shaping

Hamajima (1998) [251] proposed a manipulation flowchart for laundry folding on various garments. It includes steps from picking pieces up from a pile, garment classification, flattening and folding to putting them into the corresponding drawer. Several classical approaches have achieved this pipeline on a pile of towels [9] and even on a pile of different garments [253]. For tasks involving multiple garment types, the system needs to classify among different articles [10,51,254,255].

**Figure 5:**
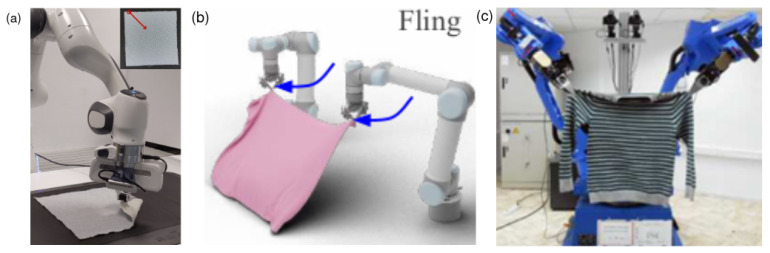
Action primitives in cloth shaping: (**a**) Pick-and-Place by Lee et al. (2021) [229], (**b**) Pick-and-Fling by Ha et al. (2022) [227] and (**c**) Gravity-based cloth flattening by Doumanoglou et al. (2014a) [10]. Figures reproduced with permission.

Humans tend to identify key points, such as corners and edges, on the article to perform cloth-shaping tasks. Sometimes, we take advantage of gravity, air dynamics and the inertia of the cloth to flatten it. Before 2018, there were two main streams of research in cloth flattening: gravity-based and pick-and-place/drag (P&P) approaches (see Figure 5). For removing the final wrinkles on the article, the system can still use P&P [256], but spreading is a more effective action primitive [253], where one arm fixes the cloth from sliding and the other sweeps to an effective direction.

Gravity-based cloth-flattening action primitives stretch the article in the air and place it on the table [50]. This requires dual arms to complete the task, where the first gripper picks up the article from a visible point or the highest point and the second grasps a point suggested using a heuristic method. The system needs to find a pair of key points on the contour of the article that can be grasped to stretch without any misgrasping and twisting [9,10,74,254,257].

Pick-and-place/drag action primitive achieves cloth shaping on the table, which can be performed by only a gripper. The difference between pick-and-place and pick-and-drag is that the second set of parameters for pick-and-place is place position, while for the latter, it is the displacement vector. Finding effective key points based on existing wrinkles [256,258,259,260] and folds [261] is crucial for flattening an article on the table.

P&P cannot erase wrinkles efficiently when the system has nearly reached its goal. In contrast, dynamic manipulation leads to a much quicker feedback cycle and self-correction on failures. Moreover, it can exploit the physical properties of the CDO to reduce the total operation time [29,226,227,228], especially for the cloth-flattening task. Apart from velocity control, one can also use inertia [227] and air dynamics [232] to control the cloth. Additionally, flipping board [51], *Japanese method* [262], a gravity-based folding method *g-fold* [252,263] and P&P action trajectories [264] are employed to perform cloth-folding tasks, where the contour of the cloth is often extracted [252,253,265,266].

Almost all RMSs that manipulate the cloth on a table apply top-view cameras, with most using depth sensors, thus capturing most of the information about the fabric while it is resting on the table. Another benefit of using a depth camera is that it is colour-invariant [256]. Optical flow provides a good representation of the relationship between the current and the goal image [237]. Mesh representation of the cloth can bring topological information into planning for grasping and flattening, and it plays a significant role in keeping track of the state of the article, especially when there are occlusions in the visual input [179,235,267].

#### 6.1.2. Data-Driven Control in Cloth Shaping

Most data-driven systems adopt classical P&P actions defined on the pixel space, and discretising P&P action space is a common technique in CDO literature. For example, Lee et al. (2021) [229] discretise the drag strategy by putting different orientations and pulling distances into bins; even though such action space simplifies the problem and leads to better training, it is less flexible during run time and harder to generalise to other tasks. Another technique that leads to better P&P policy learning is the separation of the inference of pick-and-place positions. Wu et al. (2019) [225] suggest using *Maximum Value of Placing* to select the best picking position after choosing the best placing position. Weng et al. (2022) propose *Fabric Flow Net* that infers the pick-condition place policy [237], where the pick position is an input for the place policy function.

Cloth flattening and cloth folding can be trained jointly using a uniform goal-conditioned data-driven approach [38,226,229] owing to their identical underlying dynamics as well as the similar state, action, goal and policy representations. Some methods [34,226,228,229] train MFRL agents with *HER* [182], a self-supervised technique to train agents in a sparse reward environment and generalise across the task family, to improve the data efficiency. Others [38,233] leverage the difference between current and goal observation to generate reward signals for MBRL methods. Although Yan et al. (2020) [233] only focus on the cloth-flattening task, the objective of minimising the distance between the current and the goal latent state can apply to all cloth-shaping tasks. Arnold et al. (2021) [235] and Hoque et al. (2022a) [38] attempt to apply this goal-conditioned policy representation at the mesh level with the attempt to solve more complex cloth-shaping tasks. In addition, some of the mentioned BC methods train a goal-conditioned *Transporter Network* [34,104,234] or flow network [237,268] individually to improve data efficiency, yet with P&P action primitives.

The complex and enormous state–action dynamic of the cloth makes it hard to train a policy representation in BC, a dynamic model in MBRL and a value function in MFRL algorithms. It often requires training the model with a large amount of data (more than 100k observation–action pairs) [38,233,234]. Demonstration data [20,34,38,226,236], corner-biased data [38,237] (which biases the picking action towards corners and edges of the fabric) and other engineered data collection strategies [179,235] are utilised to speed up the training. Whilst only Lee et al. (2021) [229] collect real-world data, most learning-based methods operate in simulated environments, where some apply domain randomisation [81] to achieve *Sim2Real* [38,227,233,237]. Data augmentation functions, such as scaling and rotation on the observation, are used to improve data efficiency for end-to-end cloth-shaping control systems [229]. Action noise can also be applied, but only with small perturbations, as the next state of the cloth is susceptible to the applied action [229]. Domain randomisation, such as randomising the background colour of the table [37,233], is used for transferring the simulation-trained end-to-end policies to the real world (Section 3.4).

The application of MBRL in cloth-shaping tasks suffers significantly from model exploitation and compounding error. Ma et al. (2021) [234] propose *G-DOOM*, a graph-based dynamic model based on key points to reduce compounding error, while Yan et al. (2021) apply *Contrastive forward modelling (CFM)* [233] to train latent dynamics for planning. Hoque et al. (2022a) [38] propose *Visual Foresight Modelling* that leverages variational video prediction models *SV2P* and *SVG* within the framework of *Visual MPC* [175] for mitigating model exploitation. The *Deep Planning Network (PlaNet)* latent dynamic model by Hafner et al. (2019) [156] has been examined in detail in the literature [29,233,234], but its results on CDOs are not as good as in rigid-object continuous control domains—the reconstructed observation from the visual model is fuzzy [38,233], which makes planning based on reconstructed vision hard due to the unclarity of the edges and corners of the article. Lin et al. (2022) [179] also argue that learning a latent representation loses the detailed information of the target cloth such as folds and wrinkles. Moreover, particle-wise learning-based dynamic models, such as *MeshGraphNets* [269] and *GNS* [270], achieve incredible results using GNN. *Visible Connectivity Dynamics (VCD)* [179] applies such mesh models on the visible part of the cloth to achieve more precise planning.

To tackle the exploration problem, some data-driven methods use key points of the fabric as the observation space [20,226,234] to shrink the exploration space. In contrast, others attempt to reconstruct the corresponding mesh to guide the manipulation [38,179,235]. Additionally, policy noise [156], demonstration data [20,271], specially engineered data [237,272], *HER* [182], MaxEnt-RL objective [29,225,228] and advantage-weighted loss exploration term [271] are leveraged to improve the sampling efficiency of DRL applications in cloth shaping.

In robotic applications using RL, the reward and the goal signal of cloth flattening are usually assigned based on the coverage area of the fabric in its vision input [38]. Rewards in cloth folding are usually based on the difference between the current and the goal vision inputs [38]—this helps both MFRL and MBRL algorithms train successfully [38] on simple cloth-shaping tasks. On the other hand, *CFM* [233] and *G-DOOM* [234] leverage the distance between the current and goal latent representations. Furthermore, particle-wise distance estimation from the observations between the current state and the goal state shows improved performance in two-step folding tasks [38]. Hoque et al. (2022a) [38] propose an effective dense reward function based on the coverage difference between two consecutive states for the cloth-flattening task; their method also rewards performance when reaching 92% coverage and penalises the misgrasping failures and out-of-boundary scenarios. The reward and goal signals of some MSCF tasks, such as triangle folding and square folding, cannot be directly extracted from vision input due to the self-occlusion, although we can divide them into smaller subtasks to adapt to vision signals [237].

Several benchmark environments have been proposed to accelerate the development of RMSs on cloth shaping. Lin et al. (2021) [29] created *SoftGym* based on *Nvidia Flex* [26], which includes rope stretching, cloth-flattening, cloth-folding and cloth-placing task environments—they also provide the learning performance of *Oracle MPC*, *SAC-DrQ*, *PlaNet* and *SAC-Curl* using velocity control signals as action space. Seita et al. (2021) [34] propose *DeformableRaven* based on *PyBullet*, and provide execution performance of a goal-conditioned *Transporter Network*. Hoque et al. (2022b) [272] propose a real-world cloth-flattening and cloth-folding benchmark based on *GoogleReach* [273], which is a collection of remote physical workcells, each equipped with a robot arm and a table, which can be accessed and programmed through the internet.

Human perception is mainly used to assess whether a physical trial is successful in the cloth-shaping literature, as few standard automatic metrics can evaluate the performance, especially in the real world. The mean and standard deviation of particle-wise distance towards its goal have been used in many cases [237], but these mainly apply to simulations and need to keep track of key points of the article in real-world assessments. The difference between current and goal observation has also been utilised [233,272]. It is beneficial for automating the evaluation in physical trials, but it cannot capture all information in more complex problems than one-step folding tasks. The return value of an episode [29] and the reward of the last episode steps can measure the performance of a method; however, as the reward functions of those methods differ, it is not easy to compare various methods. For cloth-flattening tasks, normalised improvement [179] and normalised coverage [38,179] are the most reliable metrics that can be automated both in simulation and reality; however, this metric does not explicitly include information on wrinkles on the cloth, which is crucial for evaluation when a trial is nearly successful. Lastly, the number of steps and inference time are used to measure a system’s effectiveness as secondary metrics.

#### 6.1.3. Challenges in Cloth Shaping

Many real-world failures in cloth shaping are caused by grasping deficiencies of the system, including (1) inaccurate grasp-point estimation, (2) misgrasping the target point on the cloth, (3) grasping multiple layers of the fabric if there is a fold and (4) rigid damage on the end-effector caused by a hard surface. Simulation-trained policies are affected since modern simulators cannot accurately emulate the interaction between the gripper and the CDO [29,34,38]. Engineering reward functions and goal evaluations for MSCF tasks is difficult because the final state of the cloth in these tasks exhibits a high degree of self-occlusion; therefore, a perception system needs to reason about the complex spatial relationships between the current and goal observation in pixel space. DRL controllers often suffer sampling efficiency due to the complex and enormous state–action space of cloth shaping. Robust velocity control is still an unsolved challenge in this domain [20,29,226], although few attempt such low-level velocity control to conduct dynamic manipulation [20,29,226,227,228].

### 6.2. Knot Tying/Untying

We define the rope-manipulation (RPM) task family as tasks executed on cloth-like linear deformable objects (CLDOs) such as ropes, wires and threads. Manipulation tasks on CLDOs include grasping, shaping, knot tying, knot untying and wrapping objects. RPM also encompasses insertion and suturing, which have applications in fields such as surgical robotics and assembly lines in factories [12]. RPM also has important applications in climbing, dressing and decoration and is a set of harder problems than cloth shaping. Rope-shaping tasks, where a robot puts a target rope into a certain shape without any crossing, have similar properties as cloth shaping so we will not go into detail on rope- shaping, as they are often covered in data-driven cloth-shaping literature which we discussed in the last section. This subsection will mainly discuss knot-tying and knot-untying applications in the literature.

Knot tying (KT) encompasses (1) tying a particular knot on a single rope (knots), (2) connecting two or more ropes (bends) and (3) tying the rope to an object (hitches). The simplest and most common knot is an overhand knot. Knots for performing single-rope tying (SRT) also include double overhand, figure-of-eight, masthead, reef and sheepshank knots, as well as the bowline, bowknot and Ume-knot [274]. Knots used for connecting multiple ropes include square and sheet-end knots, as well as strop, harness and carrick bends. Standard knots for tying a rope on an object are half and clove hitches. Finally, some knots are used for decoration, e.g., sounding lines, cloverleaf knots and Ruyi knots.

KT depends on whether the rope is tied on the table or in the air. The number of end-effectors, their capabilities and the usage of extra tools can drastically change the nature of the task. The inverse process, knot untying (KU), is also a difficult problem for robotics that correspondingly covers (1) untying a particular knot on a single rope, (2) untying a knot that connects multiple ropes and (3) untying the rope from an attached object.

#### 6.2.1. Classical Manipulation in Knot Tying/Untying

Humans mainly use three fingers (index, middle and thumb) to tie knots. The skills we use are bending, twisting, holding and binding the rope [275]. We can even make knots without vision input after we hold them in our hands, and we check whether the knot is tightened using interactive perception.

Both KT and KU start from a structural representation in a computer simulation using knowledge of knot theory. The early KU approaches in topological simulation include random perturbation [276,277], annealing schedule [278], energy minimisation with gradient descent [279] and motion planning [280,281]. Wakamatsu et al. (2004, 2006a, 2006b) [275,281,282] introduce a general motion planning framework for both topological KT and KU using four Reidemeister moves [275,283] without any energy function.

Reidemeister moves (RM) are designed to manipulate a mathematical knot that is a loop with no ends: RM I achieves loop production/removing by adding/removing one crossing; RM II simultaneously adds/removes two crossings; and RM III moves a segment to the other side of a crossing. For tackling physical ropes, the fourth type of RM [282,284] is proposed to achieve rope pulling and moving operations. Additionally, rope permutation [284] that can cancel rope deformation, continuous transformations [285] that includes rope expansion and contractions, and a set of grasping strategies [275] are proposed to improve the efficiency and success of the tasks.

**Figure 6:**
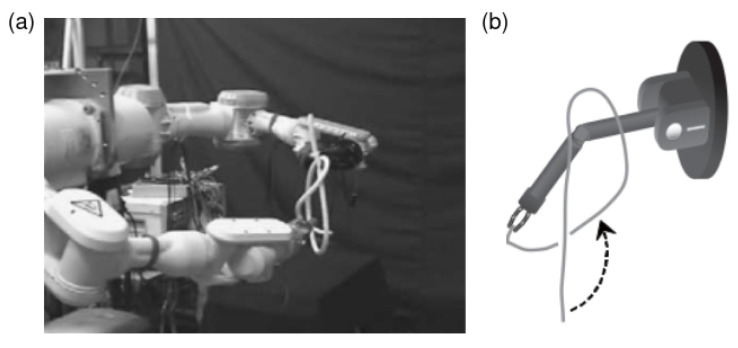
Different knot-tying tasks: (**a**) overhand knot in air by Matsuno et al. (2006) [285] and (**b**) overhand knot with dynamic control by Yamakawa et al. (2010) [52]. Figures reproduced with permission.

Most KT systems act either on the table [243,244,275,282,286,287] or in the air [52,91,105,239,284,285] as shown in Figure 6. Similar to cloth shaping, P&P action primitives are leveraged for the tasks performed on the table [286]. In KT, the agent usually requires more coordination between the two end-effectors to achieve the task. Exceptionally, Yamakawa et al. (2010) [52] achieve the overhand-knot in the air using a one-arm high-speed dynamic control system with the assistance of gravity and the rope’s inertia.

On the table, a target knot can be tied and untied by the four RMs and rope permutation that can be delivered with P&P action primitives, but this process cannot guarantee the tightness of the knot. Pulling both ends of a rope is enough for tightening simple knots such as an overhand knot. This can be achieved by either fixing one end of the rope and pulling the other or grasping both ends and pulling them towards opposite directions. However, many complex knots used for connecting and decorations must be tightened at specific locations along the rope [288] by leveraging friction locks [289,290,291,292], i.e., locations where friction resits the rope against external forces.

Most approaches in the KT/KU literature choose to operate at the topological level, so the perception system needs to extract a topological representation (TR) from its sensory data. There are many types of TR used in the robotic literature, such as P-data [61,293] (a collection of segments and intersections), K-data [61,293] (an ordered vector of intersections and segments from start to end points) and a sequential representation [275,282]—a combination of the K-data and crossing information of the 3D-to-2D projected rope with (u,l) representing crossing over or under and (+,−) [294] representing two types of crossings related to the direction of the two segments at the crossing. The common strategy for producing TR is to use the extracted intersection and segments of the rope from thinned binary images [61,105,275,282,284,286].

#### 6.2.2. Data-Driven Manipulation in Knot Tying/Untying

Apart from motion planning as described in Section 6.2.1, LfO is another line of research in KT/KU literature. Morita et al. (2003) [61] introduce *Knot Planning form Observation (KPO)* which integrates control methods from LfO on the topological level of the rope, where the changes of the representation in the consecutive demonstrated observation produce a primitive action. Sundaresan et al. (2020) [250] achieve 66% real-world success in tying the overhand-knot by improving the descriptor perception system—it can tell the exact location of the individual part of the rope in the image—within the LfO framework.

BC is also a popular method in this domain. Vinh et al. (2012) [91] use trajectory replay to achieve single-arm overhand-knot tying in the air. Kudoh et al. (2015) [105] also adopt a similar method to type a square knot on a cylinder using three-fingered dual arms. Lee et al. (2014) [243] and Huang et al. (2015) [244] employ a trajectory BC method *TPS-RPM* [117] to transfer the demonstration policy after registering current observation to a keyframe, followed by trajectory optimisation to refine the suggested trajectory. They achieve tying an overhand knot using dual arms on the table. Takizawa et al. (2019) [224] apply the same method to achieve an overhand knot and a figure-eight knot using a pair of three-fingered arms. Suzuki et al. (2021) [239] leverage the multi-modal deep BC method to achieve a 95% success rate on in-air dual-arm bow-knots and overhand-knot tying in real-world trials.

For approaches that leverage IL, the perception systems produce a rope descriptor that annotates the corresponding locations on the rope among different inputs. Sundaresan et al. (2020) [250] use a supervised learning approach with NNs to learn such descriptors. *TPS-TT* systems have a perception system that takes the current and goal image and produces a warp function that maps the corresponding points on the rope [116,243,295]: Lee et al. (2014) create such a function with registration from point cloud data [243]; and Huang et al. (2015) [244] improve perception by adding RGB imagery as input and key-point segmentation as an intermediate representation. In contrast, Suzuki et al. (2021) [239] train an autoencoder to learn the latent representation from RGB input and tactile sensors.

In terms of knot untying, Granne et al. (2020) [240] create a *Hierarchical Untangling from Learned Keypoints (HULK)* system that suggests grasping points for untying action primitives. Sundaresan et al. (2021b) [36] propose a robust rope grasping system called *Local Oriented Knot Inspection (LOKI)* that is trained with behaviour cloning using synthetic data generated from Blender [35]. Sundaresan et al. (2021b) [36] developed *Sensing Progress in Dense Entanglements for Recovery Manipulation (SPiDERMan)* to recover from the error caused by *HULK*. Viswanath et al. (2021) [241] managed to untie multiple cables with their *Iterative Reduction Of Non-planar Multiple cAble kNots (IRON-MAN)* system that generates primitive actions to remove crossing on the cables. Combining *LOKI*, *HULK* and *SPiDERMan*, the system achieves an 80% success rate in real-world trials on untangling three cables.

There are many applications of IL in the KT/KU literature, but it is difficult to frame the task as MDP to develop an RL controller—one of the challenges is reward shaping. Fan et al. (2022) [43] use DRL algorithm *Deep Q-learning (DQN)* [126] that takes embedded states as input and discretised grasping points and moving directions as action. They achieve a 54% success rate on single-arm knot untying knots on a table. Their reward credits removal of crossings while penalising addition of crossings and ineffective operations.

#### 6.2.3. Challenges in Knot Tying/Untying

In addition to CDO’s complex dynamic and self-occlusion issues, manipulation of CLDOs also suffers from another problem called self-symmetry. It means that a rope looks exactly the same from the start to the end points and vice versa. RMSs in the literature usually use a topological representation that specifies the target rope’s start and endpoints, but it is hard for a perception system to consistently keep track of the two points due to the self-symmetrical property of a CLDO. Note that self-symmetry is not a problem for cloth shaping because the system does not have to keep track of the topological structure of the cloth to solve flattening and folding tasks.

The fundamental challenge when untying a knot is to recognise its knot structure [296]. The crossing structure of a tangled rope can be more complicated than a knotted rope [281], which makes the search space of motion planning algorithms much larger. Furthermore, connecting multiple ropes introduces more crossings while trying to tie/untie multiple ropes [241] than dealing with a single rope. It also presents more endpoints of ropes in the scene, which amplifies the self-symmetry problem and makes it difficult to keep track of the status of every specific rope.

### 6.3. Dressing

Dressing tasks are the second most investigated topic in CDO manipulation literature. *I-Dress* [297] is a long-standing ongoing project that aims to develop an autonomous system to help humans with reduced mobility to dress. Self-dressing is another challenging domain that belongs to the dressing task family. A virtual self-dressing agent can provide autonomous dressing animation for film production. Although there are no significant real-life applications of self-dressing agents, they share many properties with assistive dressing systems in terms of representation and motor skills.

**Figure 7:**
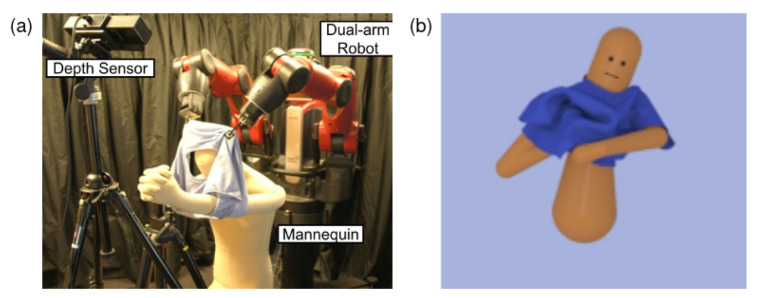
Dressing Tasks: (**a**) Assistive dressing by Koganti et al. (2017) [300] and (**b**) self-dressing by Clegg et al. (2018) [32]. Figures reproduced with permission.

**Assistive dressing** describes helping an immobile or partially immobile person to put on or take off various garments while ensuring the person is mentally and physically comfortable (see Figure 7a). The garments include hospital gowns (without sleeves), jackets (with sleeves), T-shirts, trousers [298], hats [299] and other types of garments. Moreover, the assisted person can cooperate with the agent to finish the dressing. Many older adults have limited limb movement, and some patients may even shake unpredictably during the task.

**Self-dressing** refers to putting on and taking off various garments on a humanoid robot without damaging the robot’s body and the garments (see Figure 7b). When humans put on a T-shirt they mainly rely on their body’s haptic system. For example, they may first put the bottom of the T-shirt over their head, then find and put the corresponding sleeves over their arms, put the head through the hem, and finally pull down and adjust the garment. While stretching our arms through the sleeves, we do not think about the complex interaction between the article and our limbs [32,230]. At the same time, we are careful not to get snagged in or tear the cloth.

#### 6.3.1. Classical Manipulation in Dressing

Assistive dressing is a long-horizon multi-step task that requires safe and reliable human–robot interaction. For instance, while helping a person put on a jumper, the agent needs to tuck the person’s head into the jumper’s hem, put the two individual arms into the corresponding sleeves, and then pull down and adjust the jumper. These subtasks, though they need to be executed sequentially, are usually investigated individually for various types of garments in the literature. When a human helps another person to dress, they can use life experience to estimate the force exerted on the assisted person [55]. Similarly, we expect the system to provide smooth, predictable action trajectories exerting small forces, to react accordingly to human posture and motion and to avoid damaging the cloth.

A common approach of assistive dressing is to combine collision avoidance and compliant control [86] by leveraging a motion planner [299,301,302] with/without posture tracking models [298,303,304] to generate a collision-free [305] predictive action sequence, then adopt compliant control with force sensors [306], or distance control [307], to move relative to body posture [308]. Keeping track of the body–cloth relationship is essential for successful and safe assistive dressing. Topological coordinates [1], where a skeleton structure represents the torso [298,309] and circles represent the opening part of the article, are often adopted for reasoning such relationship. Robust real-time estimation [300,310,311,312] of such topological relationships is essential for real-world trials.

Such skeleton extractions and posture tracking can provide trajectories that avoid collisions between the end-effector and the body. We still need reliable force estimation [32,55] for safe and comfortable reactive manipulation. Force sensors can help mitigate occlusion problems in visual perception [73,303,304,313,314]. In addition, the system needs to detect success and different error states [298,301,309,315,316,317]. The ability to recover from failures without repositioning the target human [298,309] is equally significant.

#### 6.3.2. Data-Driven Manipulation in Dressing

In the data-driven control domain, the assistive dressing literature tends to use the trajectory BC (Section 4.3) methods to generate initial trajectories then utilise Type I MBRL (Section 5.3) methods for safe and smooth execution of trajectory. Tamei et al. (2011) [1] adopt an RL framework [318] to tuck a mannequin’s head into a T-shirt’s hem and its arm into T-shirt sleeves using dual arms; they initialise the “via-points” with demonstration trajectory and refine the trajectory using the policy gradient method [319]. Matsubara et al. (2013) [246] use the same RL framework for learning self-dressing T-shirts on a dual-arm robot, specifically, putting both arms into the sleeves of the T-shirt. Colome et al. (2015) [247] use *Dynamic Movement Primitive (DMP)* [111,112] to initialise the robot trajectory to wrap a scarf around a human’s neck using a single arm; they use *PI2* [320] to refine its policy. Pignat et al. (2017) [248] formulate the task where a robot assists a human to put their arm into a jacket with *Hidden Semi Markov Model* (HSMM) [107] for encoding the demonstration trajectory using *EM* algorithm, and they leverage a *LQR* [109] to drive the robot to follow the generated trajectory from the forward messages of the HSMM [321]. Joshi et al. (2019) [249] break down the task into three consecutive subtasks and apply *DMP* for dressing the arm and a *Bayesian Gaussian Process Latent Variable Model* (BGPLVM) for dressing the body. In the application of trajectory BC, the real-world demonstration trajectory is usually collected by directly controlling the robot arms with hand, known as kinesthetic teaching [1,246,248,248].

On the other hand, several attempts focus on using a pure RL controller. Clegg et al. (2017) [230] use the policy-gradient method *Trust Region Policy Optimisation (TRPO)* [134] (Section 5.1.2) with curriculum learning [322] in simulation to learn a modular haptic feedback controller of self-dressing. The same team [32] then manage to learn a complete self-dressing task in simulation with a specially engineered reward function (2018). The observation space includes the human’s joint angles, garment feature locations, haptics, surface information and a task vector. They later employ the same method for training a simulated dual-arm robot and a human with various motor capabilities for wearing a hospital gown and T-shirt collaboratively [31]—the observation includes sensorimotor information of both robot and human, force signal from the end-effector and target pose from the previous time step; the action space in both approaches is based on the positional signals of the joints [31,32]. In a real-world trial, this system requires the human to wear sensory equipment on their body. They achieve *Sim2Real* transfer by calibrating the simulator with real-world data and scaling down the policy output.

Reward engineering is a hard problem in the dressing domain. Tamei et al. (2011) [1] and Matsubara et al. (2013) [246] design their reward function around the topological coordinate (comprising writhe, centre and density) distance between the configuration of the region of interests of the torso and the garment. Colome et al. (2015) [247] suggest a reward based on penalising high acceleration and high estimated force. They also included another term indicating how well the scarf is placed in images. Clegg et al. (2017) [230] penalise the distance to the goal and the failure of the task. In 2018, the same team suggested a self-dressing reward function that comprises progress reward, deformation penalty (for avoiding tearing the garment), geodesic reward and per-joint "rest pos" reward (where the user body is in the default setting) [32]. However, this reward is not safe for the human body in assistant dressing tasks, so they added a further term to reduce the force received by the human [31].

Conventionally, evaluation of assistive dressing is conducted on a mannequin during development time and on real humans for final evaluation [301]. Self-dressing can be developed and tested on the robot itself in the real world. [246]. However, such physical testing cannot provide a large volume of data for a confident conclusion of robust manipulation. Hence, simulation becomes an ideal place for developing and testing dressing approaches [31,55,317]. The most common simulator adopted in this domain is *Nvidia PhysX* [30]. Furthermore, the mannequin cannot provide unexpected or collaborative actions in development and final testing. Clegg et al. (2020) [31] suggest using simulation to create scenarios where humans have different levels of disability: dyskinesia, limited range of motion and muscle weakness. In the real-world trial, they employ another humanoid robot to replace a mannequin for creating unexpected and collaborative motions.

#### 6.3.3. Challenges in Dressing

The ability to distinguish the inner surface of an article from the outer helps to avoid becoming tangled in the article [230]. In self-dressing, it is specifically challenging for an agent with a tactile sensor to formalise and integrate such perception ability into the control procedure [32]. In general, self-dressing is easier than assistive dressing, but the two share some common challenges.

First, the topological and functional properties of garments are highly correlated [323]. Reliably finding the topological correspondence between the different parts of the body and the garment is a challenge for perception [246,312]. The problem mainly lies in the occlusion of the garment by the torso and the deformation of the article itself [300], where such deformation can be quite different when compared to cloth-shaping tasks [300]. Second, finding the effective grasping point and grasping strategy for dressing the corresponding parts is still an underexplored problem. Almost all of the literature features experiments with the presumption of grasping a correct part of the article, while only Clegg et al. (2018) [32] integrate grasping as part of the skill learning using DRL. Third, dressing tasks generally deviate from common manipulation tasks. They heavily depend on tactile and haptic sensors to infer the progress of the task [32]: (1) force estimation of the cloth on the body is crucial for safe and comfortable dressing; (2) estimation of the cloth-stretching degree helps to avoid damaging the cloth. It is not obvious how humans leverage such perception to perform a similar task, and this remains a difficult open problem in this domain [230].

In addition to the challenges mentioned above, the agent should also react appropriately to cooperation and the unpredictable motion of the user in assistive dressing. Unexpected user movements may lead to dressing failures or even pose risks to the user. The complex deformation of the cloth and its occlusion on the body makes human-posture tracking difficult [31,304]. For example, occlusions can occur when the robot’s arms, the garment and the human body are in close contact [306]. Such occlusion makes it hard for the vision system to accurately observe the task state and predict the results of planned interactions. Furthermore, it is hard to test assistive dressing in the real world during the development stage, as mannequins cannot be easily actuated [249].

### 6.4. Bag Manipulation

Bag manipulation is a relatively new and less investigated domain among the four task families in the literature, so we will cover classical and data-driven manipulation methods together in this section. We characterise bags as 3D cloth-deformable objects that can contain items. More specifically, bags refer to 3D CDOs that have handles, while sack represents bags without handles. RMSs with bag-manipulation skills could help a human with grocery shopping and transporting heavy items. We note that robotic applications on bags with handles are rare compared to sacks.

**Figure 8:**
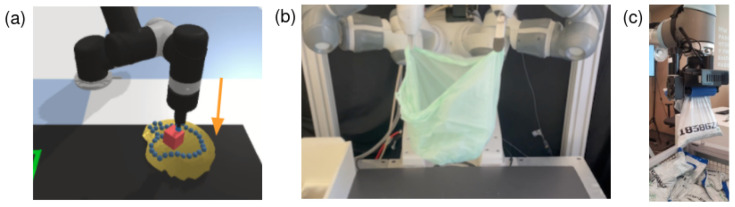
Different bag manipulation tasks: (**a**) Item insertion by Seita et al. (2021a) [34], (**b**) item containing/sack lifting reproduced by Seita et al. (2021b) [242], and (**c**) sack moving by Gonnochenko et al. (2021) [231]. Figures reproduced with permission.

The canonical task of bag manipulation is item loading [238] which involves opening the bag, inserting items [34] (see Figure 8b) and lifting the bag. Lifting a bag with an item in it is also known as an item containing [242] (see Figure 8b). The general grasping ability of various items and the effective grasping strategy of the bags are crucial for the success of item loading. Item inserting can be more complex if there are other bags for distraction [34]. The next common task is bag moving [238] (see Figure 8c), which includes lifting the bag, creating displacement and placing the bag at the target position. The third standard task is item unloading, which requires opening the bag and picking out the items.Finally, bag unloading [231] involves the agent unloading a collection of bags from a basket.

#### 6.4.1. Classical and Data-Driven Manipulation in Bag Manipulation

To perform item loading, two end-effectors are required to firmly grasp the appropriate point on the opening of the sack [242] or find the handle of the bag. Seita et al. (2021b) [34] adopt a combination of heuristics and imitation learning to learn the grasping position on a sack. The heuristic strategy is to grasp the endpoints of the maximum width of the sack mask. They also leverage data augmentation (rotation and translation) on the current and goal images to improve the representation capability of the system. Xu et al. (2022) [232] propose a system called *DextAirity* that uses air-blowing to open a sack while grasping the hem of the sack with bilateral grippers.

To develop a system that can conduct item containing, Seita et al. (2021b) [242] produce a sack mask from a top-down depth camera for generating grasping candidates for a dual-arm robot. They also leverage interactive perception for checking if the target bag/sack is grasped robustly—the robot applies a shaking motion to test whether the bag will slip or not [242].

In bag unloading, Kirchheim et al. (2008) [324] and Gonnochenko et al. (2021) [231] infer the depth of individual sacks from a depth sensor using detection and segmentation techniques. They also employ a roller end-effector to perform sack unloading. Kierchheim et al. (2018) apply a handcrafted policy for effective grasping, while Gonnochenko et al. (2022) adopt DRL to learn the grasping position and orientation in the *Mujoco* simulator.

The benchmark environment *DeformableRaven* [34] includes modular sack manipulation environments. Seita et al. (2021a) [34] adopt a *Transporter Network* [104] to learn a goal-conditioned policy for sack opening and item insertion tasks on this benchmark. Teng et al. (2022) [238] add a dense network layer [325] and residual connections [326] in the transporter net to enhance feature extraction, and they further examine the system on item loading and bag moving. Both approaches train the policy network only with successful trials in simulation induced by a scripted demonstration policy. In simulation, Seita et al. (2021a) [34] achieve a 63.3% success rate out of 60 trials for sack opening and a 51.7% success rate for inserting one item in simulation. Teng et al. (2022) [238] achieved a 48.3% success rate out of 60 trials for item loading and sack moving in simulation.

#### 6.4.2. Challenges in Bag Manipulation

Compared to ropes and cloth, deformation of bags is more complex, and self-occlusion is much more severe for an RMS [242]. The complex dynamic between the rigid/deformable items with the bag [42] gives another layer of challenge for the item loading task. During item insertion, the perception needs to reason whether the objects are within the region of open contour [238,242] of the bag. While conducting item containing, the system also needs to reason whether items will remain inside the bag under their interaction with the bag and gravity [242]. Effective grasping strategy on a sack, which takes advantage of gravity and other factors, is still an open problem in this domain [242].

Lifting a bag with items inside it demands an accurate weight estimation so that the bag does not slip from the gripper, as is common when grasping a thin layer [242]. Sacks introduce further difficulties compared to bags as they do not present handles. They usually require special end-effectors, such as rollers [231], so the agent can effectively grasp and hold the sack without damaging the sack. Moreover, bags can have different shapes and sizes, and the centre of gravity changes while transporting the bag. The agent also needs to consider these factors for safe performance [231].

## 7. Discussion and Future

Cloth-like deformable object manipulation is a very active research area in robotics, and grasping is a major common challenge in all CDO manipulation domains. Robust grasping in CDO refers to the accurate grasping of the target point without misgrasping or grasping the wrong layer of a folded CDO. Additionally, robust grasping demands persistent holding of the target point without damaging the CDO while performing a certain subtask. A robust RMS should be able to recognise the failing states and recover from these failures [9,36]. Most of the failures in the cloth-shaping literature are a result of the deficiency of robust grasping. The main reason the simulation-trained system fails is that none of the simulators provides accurate collision and friction modelling between the gripper and CDOs. Robust grasping has largely been unexplored in the dressing domain.

Apart from robust grasping, the success and efficiency of CDO manipulation highly rely on effective grasping. This means that the system should target an effective point to grasp to accomplish a certain skill or a subtask. This usually introduces inductive biases to individual task domains—(1) the corners and the contour of the articles are crucial for cloth-shaping tasks; (2) a set of grasping strategies [281] for different topological scenarios for knot tying and untying tasks are proposed to accomplish a certain move; (3) bag manipulation systems focus on the opening contour and the handles; and (4) the dressing literature often ignores this problem by letting the agent pregrasp an assigned key point before executing a task, and it remains an important uninvestigated research area for achieving a fully automated dressing agent. The grasping strategy is the first type of inductive bias in these four domains. A general agent should be able to flexibly adjust its grasping strategy based on the article type and the goal of the task, which can be delegated to individual motor skills.

The application of reinforcement learning has been heavily investigated in the cloth-shaping and dressing domains. There are fewer DRL applications in dressing and none in the bag and knot tying/untying literature. Reward shaping is one of the obstacles to applying RL to the CDO domain. Although there have been several attempts [1,31,38], designing a simple and effective dense reward function for cloth folding and dressing tasks is still challenging. Moreover, we are aware of no literature on solving knot tying/untying using RL, probably due to the difficulty in defining a dense reward function. Sparse-reward settings may help, but detecting the success of a particular task/subtask automatically is equally difficult.

The second inductive bias is the difference in the reward functions between the four task families. A promising solution to avoid reward shaping in CDO is to use IL that has been investigated in detail in all four domains. However, these applications are mostly based on behaviour cloning (BC) and learning-from-observation (LfO) methods. We are unaware of approaches based on inverse reinforcement learning (IRL), a type of data-driven imitation learning method. The recent advancement of Adversarial IRL shows substantial improvements compared to BC baselines in continuous control settings [68]. This could be the key technology to bypass the complex reward engineering in the CDO domain while preserving data efficiency similar to DRL methods. Furthermore, LfO with IRL is probably most similar to how humans attempt to imitate the demonstrator’s intention; it will be interesting to see the application of such approaches in the CDO domain.

The differences in the intermediate representations and perception systems among the four domains account for the third and the fourth inductive biases: (1) most cloth-shaping systems keep track of the contour and corners of the cloth, while some attempt to reconstruct the mesh representation; (2) most knot tying/untying systems leverage topological representation of the rope, while some keep track of the individual points on the rope; (3) bag manipulation systems are mainly interested in the openings and handles as well as the size and shape of the bag; and (4) the dressing systems are mainly interested in the relationship between the article and the body, as well as keeping track of human motion for safe and reactive manipulation. Similar to the grasping strategy, the variation of perception systems can be delegated to individual motor skills. Many approaches also attempt to learn a latent representation in self-supervised and unsupervised manners for generalisation. The representation-learning community in DRL and DIL can take inspiration from the challenges of perception in these four domains to propose a more general and effective representation-learning algorithm. Moreover, applications of transfer learning, multi-task and continual learning in the CDO domain are interesting directions to explore.

To develop a robust RL skill controller, we cannot avoid the exploration component because the agent needs to encounter different scenarios to learn to achieve goal states from them. SOTA RL exploration strategies have not been commonly applied in any of these domains. The major obstacle for the exploration is CDO’s large state–action space and the complexity of the state–action dynamic. It will be interesting to see if the SOTA exploration strategies can improve the data efficiency of RL in CDO domains. Skill-level environments of CDO could also be a good development benchmark for the progression of DRL and DIL, where CDO sets challenges to exploration and state estimation for these data-driven methods due to its complex state–action dynamic and severe self-occlusions.

Multi-modal learning in DRL has not been explored much in mainstream research. In reality, humans are highly reliant on haptic sensors to control the force and infer the material property of objects, in addition to vision. In the CDO domain, we can tie a knot without looking at the rope, and we depend on the haptic sensor to dress and even help others to dress garments. Furthermore, we can roughly estimate how much force we can exert on a certain object. Building an observational world model that incorporates both vision and haptic signals is an interesting research direction for robotics development.

### Summary

This review has covered the state-of-the-art developments in four task families in CDO manipulation, including cloth shaping, knot tying/untying, bag manipulation and dressing. Most systems focus on skill developments, where each task domain is beginning to adopt data-driven approaches such as DIL and DRL to achieve more robust and general skill controllers. Attempts at solving long-horizon multi-step tasks that involve multiple articles and other items are rare in this field. It will be beneficial to build benchmark environments for tasks such as doing laundry [9,251] and a full set of assistive/self-dressing/undressing tasks. We expect that this will require the involvement of hierarchical control methods based on the frameworks of *options* and *semi-MDP* [327].

We identify four types of inductive biases that occur in the four task families: differences in grasping strategies, reward engineering, intermediate representations and perception systems. We also outline the recent advancement and tools of robotics, DIL and DRL that can be employed in CDO manipulations, along with their challenges so that readers will be aware of these obstacles while applying them. Lastly, we summarise the future direction of applying data-driven methods in CDO manipulation. We hope this review can provide a comprehensive guideline for the progression of the field. 

## Figures and Tables

**Table 1:** Overview of data-driven CDO literature.

	Cloth Shaping	Knot Tying/Untying	Dressing	Bag Manipulation
MFRL	[20,29,225,226,227,228,229]	[43]	[31,32,230]	[231,232]
MBRL	[29,38,179,233,234,235]	-	-	-
State-action BC	[34,236,237,238]	[36,239,240,241]	-	[34,238,242]
Trajectory BC	-	[91,105,243,244,245]	[1,246,247,248,249]	-
LfO	-	[61,250]	-	-

## Data Availability

Not applicable.
